# Mixed methods systematic review and metasummary about barriers and facilitators for the implementation of cotrimoxazole and isoniazid—Preventive therapies for people living with HIV

**DOI:** 10.1371/journal.pone.0251612

**Published:** 2022-03-01

**Authors:** Pia Müller, Luís Velez Lapão

**Affiliations:** Global Health and Tropical Medicine, Instituto de Higiene e Medicina Tropical (IHMT), Universidade Nova de Lisboa (UNL), Lisboa, Portugal; University of Washington, UNITED STATES

## Abstract

**Background:**

Cotrimoxazole and isoniazid preventive therapy (CPT, IPT) have been shown to be efficacious therapies for the prevention of opportunistic infections and tuberculosis (TB) among people living with human immunodeficiency virus (HIV). Despite governments’ efforts to translate World Health Organization recommendations into practice, implementation remains challenging. This review aimed to explore and compare CPT and IPT with respect to similarities and differences of barriers identified across high TB/HIV burden countries. A secondary objective was to identify facilitators for implementing both preventive therapies.

**Methods:**

We searched MEDLINE, Web of Science and SCOPUS databases for peer-reviewed literature published before September 2020. We extracted and synthesized our findings using Maxqda software. We applied framework synthesis in conjunction with metasummary to compare both therapies with respect to similarities and differences of barriers identified across seven health system components (in line with the modified WHO’s Framework for action). Protocol registration: PROSPERO (CRD42019137778).

**Findings:**

We identified four hundred and eighty-two papers, of which we included forty for review. Although most barrier themes were identical for both preventive therapies, we identified seven intervention-specific themes. Like for CPT, barriers identified for IPT were most frequently classified as ‘service delivery-related barriers’ and ‘patient & community-related barriers’. ‘Health provider-related barriers’ played an important role for implementing IPT. Most facilitators identified referred to health system strengthening activities.

**Conclusions:**

For researchers with limited working experience in high TB/HIV burden countries, this review can provide valuable insights about barriers that may arise at different levels of the health system. For policymakers in high TB/HIV burden countries, this review offers strategies for improving the delivery of IPT (or any newer therapy regimen) for the prevention of TB. Based on our findings, we suggest initial and continuous stakeholder involvement, focusing on the efficient use and reinforcement of existing resources for health.

## Introduction

### Background

In 2020, 37.7 million people worldwide were living with the human immunodeficiency virus (HIV) [1]. An estimated quarter of the world’s population was latently infected with *Mycobacterium tuberculosis*; 10 million developed active tuberculosis (TB) [2]. The fact that HIV-positive individuals have 18 times higher risk of developing active TB disease compared to HIV-negative individuals has transformed many low- and middle-income countries (LMIC) into countries with a high dual burden of TB and HIV [2, 3]. To date, TB remains the leading cause of death among people living with HIV (PLHIV) [3]. This review focuses on the implementation of two of the most important preventive therapies for PLHIV in countries with a high burden of TB/HIV: cotrimoxazole (CTZ) and isoniazid (INH).

Besides preventing some AIDS-associated opportunistic diseases (*Pneumocystis jirovecci* pneumonia (PCP), toxoplasmosis), CTZ has shown to be successful in reducing malaria, severe bacterial infections, and mortality among PLHIV. As a result, World Health Organization (WHO) recommends cotrimoxazole preventive therapy (CPT) lifelong for PLHIV in resource-limited settings where malaria and, or severe bacterial infections are highly prevalent, irrespectively of their CD4 count. In settings were neither malaria nor severe bacterial infections are highly prevalent, CPT is recommended for PLHIV with severe or advanced HIV disease (WHO clinical stage 3 or 4, or CD4 count below 350 cells/mm^3^) and may be discontinued for those with evidence of immune recovery or viral suppression on antiretroviral treatment (ART). Adults, including pregnant women, children and adolescents with HIV, HIV-exposed but uninfected infants, and PLHIV with active TB, are eligible for CPT, including those concurrently receiving ART [4]. With its anti-mycobacterial activity, isoniazid monotherapy is prescribed to treat latent TB infection and prevent the progression from latent to active TB. WHO recommends at least six months of isoniazid preventive therapy (IPT) to people at risk of TB living in resource-constrained and high TB and HIV prevalence settings. PLHIV comprise a major risk group for TB, among which IPT has shown to reduce TB disease and mortality irrespective of receiving ART. Therefore, PLHIV, unlikely to have active TB, are eligible for IPT. This includes HIV-positive pregnant women, adolescents, infants aged under 12 months who are in contact with a TB case, and children older than 12 months who have no contact with a TB case. HIV-negative children aged under 5 years who are household contacts of people with bacteriologically confirmed pulmonary TB comprise another target group for IPT. Independent of the target group, only people with unknown or a positive tuberculin skin test (TST) unlikely to have active TB are eligible for IPT [5].

### Rationale

Data from clinical trials and observational studies demonstrated that both preventive therapies are well-tolerated, highly efficacious, and cost-effective among PLHIV [6–10], resulting in WHO recommendations that were first adopted almost two decades ago [11, 12]. With increasing evidence supporting the benefits of the preventive therapies for PLHIV, WHO recommendations have been even expanded with regard to therapy duration and target populations indicated for CPT and IPT in today’s recommendations [2, 4]. Although efforts have been made by governments of high TB/HIV burden countries and their partners to translate these recommendations into national policy and practice, implementation of both preventive therapies has been challenging [13, 14].

### Objectives

The primary objectives of this review were to explore barriers to both preventive therapies reported across high TB/HIV burden countries (as per WHO [3]) and to generate explanatory knowledge of why their implementation has been so challenging. Additionally, this review aimed to compare both preventive therapies with respect to similarities and differences of barriers. A secondary objective was to identify strategies (facilitators) to improve the implementation of both preventive therapies. To identify relevant research, the broad question: “Which are the barriers to and facilitators for the implementation of preventive therapies (CPT, IPT) in countries with a high burden of HIV and TB?” was designed using FINER criteria [15] and the PICo framework: Population, Interest, Context (modified PICO) [16] (S1 File). Barriers were defined as factors that limit, challenge or inhibit implementation, access, provision, delivery, or adherence to CPT or IPT. Facilitators were defined as factors that facilitate, support, encourage, or enable the implementation, access, provision, delivery, or adherence to CPT or IPT.

## Methods

### Protocol and registration

Following the Joanna Briggs Institute (JBI) methodology for mixed methods systematic review, we developed a protocol prior to undertaking the review [16, 17], which we registered and published in PROSPERO (CRD42019137778). We followed PRISMA guidance and reported our findings, according to the PRISMA checklist (S2 File) [18].

### Eligibility criteria

Only peer-reviewed scientific papers meeting all of the following eligibility criteria were included: (1) papers reporting barriers and, or facilitators for either or both preventive therapies (CPT, IPT), sometimes in the literature also referred to as ‘preventive treatment’, ‘prophylaxis’, or ‘prophylactic treatment’. (2) Only studies conducted in high TB/HIV burden countries defined by WHO in the period 2016 to 2020 [3]; (3) published in English language; (4) until the 4th of September 2020 were eligible for this review. (5) Studied populations eligible were: (a) HIV patients or PLHIV. Although HIV-negative population groups may be eligible for IPT (e.g. household contacts of TB cases), HIV-negative population groups were not within the scope of this review. However, for CPT, HIV-exposed babies were eligible study populations, as well as patients co-infected with TB and HIV. Other studied populations eligible were (b) healthcare providers, also referred to as health professionals; (c) caregivers; (d) any other stakeholder identified as influential in the overall implementation process of either IPT or CPT, and (e) countries defined by WHO as high TB/HIV burden countries [3]. Since this systematic review aims to analyse studies reporting primary data, we excluded editorial comments and systematic reviews. However, we screened reference lists of systematic reviews for original research eligible for this review. We included studies conducted in multi-sites or multiple countries only if barriers/ facilitators were separately analysed and reported per site or country. Because of the limited number of published qualitative studies on this topic, we included primary studies of any design (i.e. qualitative, quantitative, multimethod and mixed methods studies).

### Information sources and search

In February 2018, we systematically searched the electronic databases MEDLINE^®^, Web of Science^®^ and Scopus^®^ for original articles using the search terms presented in (S3 File). We repeated the search in September 2020 to identify additional literature published after February 2018 and updated our review [19]. The full electronic search strategy is available for each database search (S3 File).

### Study selection, data collection process and data items

Following the search, PM collated and uploaded all identified citations into Endnote X9 reference management software and removed all duplicates. We independently screened and assessed all titles and abstracts against the predefined eligibility criteria for this review. Non-relevant studies and studies reporting from countries other than the thirty high TB/HIV burden countries were excluded during this initial title and abstract screening process. When the decision on exclusion was not clear, we included the study for full-text screening. We both independently assessed the full-text of the remaining studies against the eligibility criteria. Studies that did not meet all eligibility criteria were excluded, and reasons for exclusion were recorded. Studies that met all eligibility criteria were included. We compared our decision (i.e. inclusion/ exclusion and reason for exclusion) for each of the selected studies, discussed their full-text and resolved any disagreement concerning our decision through discussion. We developed a data extraction table which we initially tested on three studies to ensure that all relevant data items could be extracted. PM extracted the following data items from each included study: first author, year of publication, geographic origin (one or multiple countries), context (e.g. urban, rural), study type, study subject(s) of interest, sample size of study subjects for each data collection approach, study aim(s), data collection approach (e.g. interviews, record review), and findings related to the review question (i.e. barrier(s) and, or facilitator(s) for CPT, IPT, or both). LVL checked the extracted data items for accuracy and added or modified data items where necessary. We compared the data we individually extracted and resolved any disagreement through discussion.

### Data transformation

As outlined in Section 8.5.1 of the JBI Reviewer’s Manual, we extracted findings (barriers and facilitators) from quantitative, qualitative and mixed methods studies. Qualitative findings, including the qualitative component of mixed methods studies, were extracted as presented in the original research paper (e.g. themes, corresponding illustrations, paragraphs of textual description). We transformed quantitative findings, including the quantitative component of mixed methods studies, into textual description disregarding the effect size [17]. Finally, we merged qualitative findings and transformed study findings together into one data set.

### Risk of bias in individual studies

For the critical appraisal of methodological quality (internal validity) of the included studies, we selected the “Mixed Methods Appraisal tool” (MMAT), version 2018 [20]. As reported by the Cochrane Qualitative and Implementation Methods Group, this tool has been used widely in systematic reviews and has the advantage of being able to assess interdependent qualitative and quantitative elements of mixed-methods research [21]. First, we independently identified the categories of study design using the MMAT algorithm and then appraised each study against the corresponding methodological quality criteria [20]. We resolved any disagreement in rating through discussion. For studies that failed to meet more than one quality criteria, we discussed whether to exclude the study. Due to the risk of excluding insights relevant for a good understanding of the phenomenon under study, which may only become apparent at the point of synthesis, bias was toward inclusion [22, 23].

### Qualitative synthesis

We applied framework synthesis, a highly transparent and deductive approach recommended for the synthesis of evidence on complex interventions [24]. This approach allows combining elements of critical realistic and subjective idealistic epistemology. We analysed our data set using MAXQDA Analytics Pro software (Release 18.1.1.), iteratively coding and sub-coding the extracted results. We both individually defined, cross-checked, discussed and refined the code system. We resolved disagreement through discussion.

Inspired by Getahun et al. (2010) [25], we applied a priori defined, slightly modified version of the WHO’s Framework for action [26] to analyse and present barriers to preventive therapies. First, we assigned each extracted result (i.e. barrier) to one of the seven health system components based on the level at which they hindered implementation. Health system components include ‘Patient & community’, ‘Health providers’, ‘Clinical information’, ‘Leadership & governance’, ‘Pharmaceutical management’, ‘Service delivery’ and ‘Financing’. We added ‘Patient & community’ as the seventh component to the existing framework. Second, we thematically analysed each barrier considering its contextual description. Third, to summarise the comprehensive barrier descriptions, we applied metasummary—a quantitatively oriented aggregation of qualitative findings first proposed by Sandalowsky, Barroso and Voils (2007) [23, 27].

For facilitators, we applied ‘the preventive therapy cascade’ as framework for evidence synthesis. After familiarising ourselves with the extracted data set, reading and re-reading the identified facilitators and exploring underlying patterns, we found that facilitators generally aimed to enhance specific activities (e.g. providers’ prescribing practices, patients’ adherence) along with a series of steps involved in the implementation of preventive therapies. Eventually, the preventive therapy cascade emerged as most relevant framework for the synthesis and presentation of our data, featuring each step along the preventive therapy cascade with all its bottlenecks. We assigned each extracted result to one or multiple steps along the cascade, followed by thematic analysis, inductively grouping facilitators into overarching themes [24].

### Risk of bias across studies

Although publication bias is primarily a concern for systematic reviews evaluating an intervention’s effect, it is debatable whether the consequences of such bias is as potentially serious in qualitative evidence synthesis [28]. More research is required on how to assess dissemination bias in the context of qualitative evidence syntheses [29]. We analysed the heterogeneity of the countries and the study subjects represented in this review and discussed potential limitations resulting from a heterogeneous representation of these variables.

## Results

### Study selection

**Fig 1:**
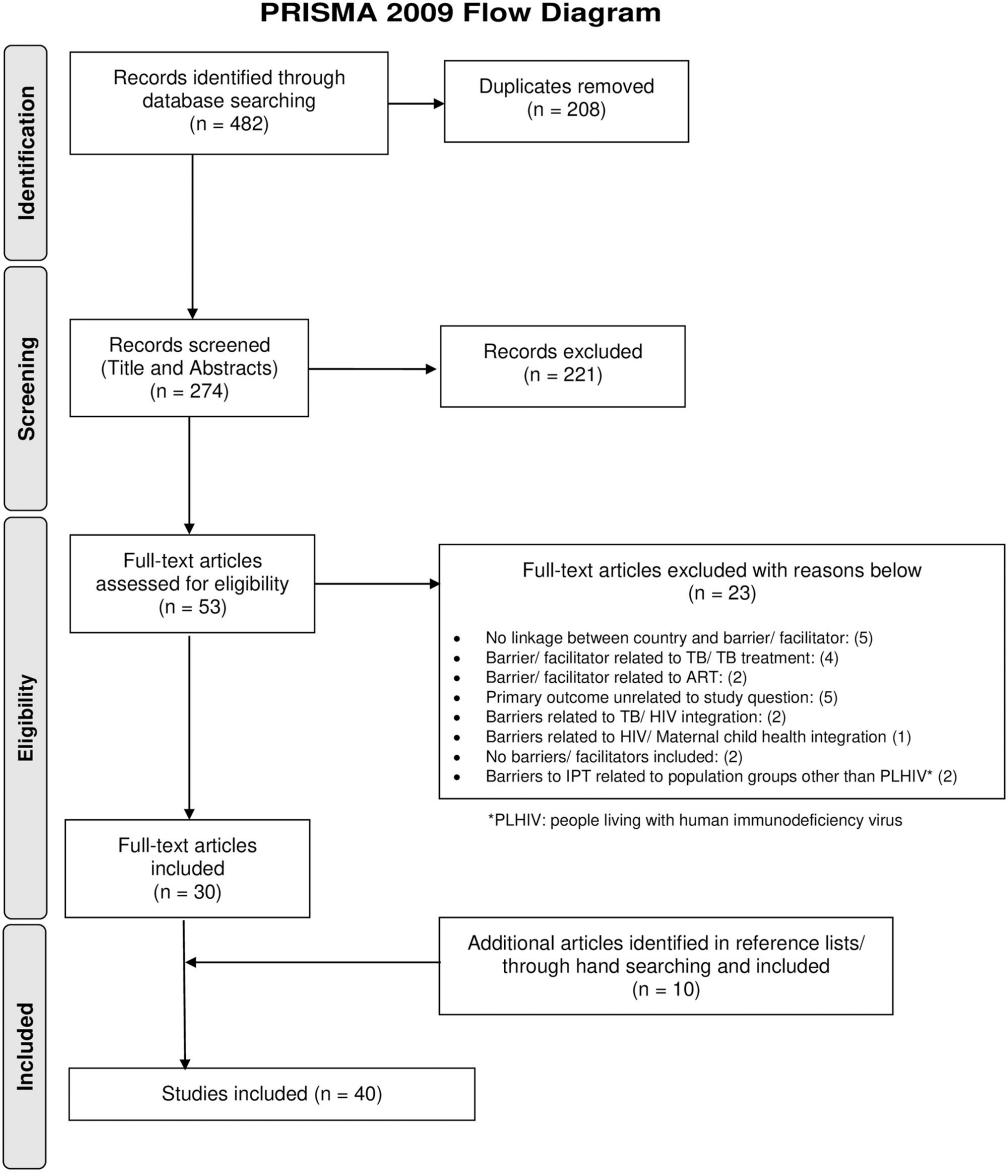
PRISMA flow diagram.

The PRISMA Flow Diagram presents the number of papers included throughout the selection process, alongside with the reasons for exclusion (Fig 1).

### Description of studies included in the systematic review

Forty studies were included for review, fourteen related to CPT [13, 14, 30–41]; twenty-eight related to IPT [13, 37, 42–67], including two studies that reported findings on both preventive therapies [13, 37]. While facilitators were reported in all studies, barriers were not presented in three studies [31, 39, 40], all related to CPT. All studies related to CPT were carried out in WHO African Region [13, 14, 30–41], while studies related to IPT also included WHO South-East Asia Region [46, 60, 61], WHO Western Pacific Region [46] and WHO Region of the Americas [45, 46].

Among the thirty countries defined by WHO as countries with a high burden of TB and HIV [3], we identified research from twenty-three countries (77%) reporting barriers or facilitators to either or both preventive therapies. Countries were disproportionally represented. The majority of studies were conducted in South Africa (n = 11) [36, 41, 46, 49–51, 53, 62–64, 66], Uganda (n = 9) [30–32, 37–39, 43, 55, 65], Tanzania (n = 5) [14, 34, 35, 46, 59], Kenya (n = 4) [44, 46, 48, 67], Zimbabwe (n = 3) [32, 33, 46], Ethiopia (n = 3) [46, 52, 57], and Nigeria (n = 3) [42, 46, 56]. Another fifteen countries were represented in only one or two studies. All studies, except three [32, 46, 48] based their findings on data collected in one single country. Among the twenty studies that reported the context, ten were conducted in urban areas [32, 34, 35, 40, 43, 44, 48, 57, 66, 67], eleven in rural areas [32, 37, 38, 41, 44, 48, 49, 54, 61, 62, 65] and six in intermediary areas [32, 37, 41, 43, 50, 64] (i.e. peri-urban, sub-urban, and semi-urban context). Some studies were conducted in multiple contexts.

Patients (n = 24) [30, 31, 34, 35, 37, 42, 43, 45, 47–51, 53, 54, 57–65], health providers (n = 14) [13, 14, 35, 37, 38, 44, 45, 50, 52, 53, 61, 62, 66, 67], and health facilities (n = 14) [14, 32, 35, 36, 38–41, 43, 44, 48, 55, 56, 66] were the study subjects of interest most frequently represented in this review, with at least one of the three study groups included in thirty-eight studies (95%). Few studies included other study subjects, explicitly caregivers [14, 33], community members [37], other stakeholders [13, 44, 45, 55], districts [37], and countries [46]. Table 1 shows the main characteristics of the studies included in this review.

**Table 1:** Main characteristics of the studies included in this review.

First author [citation]	Year of publica-tion	Country	Study type	Study subjects	Data collection approach	Treat-ment of interest
Adepoju [42]	2020	Nigeria	Retrospective cohort study	Patients	Record review	IPT
Aisu [43]	1995	Uganda	Operational Assessment	Patients, Facilities	Clinic attendance monitoring, Adherence (Pill count), Interviews, Review of key documents	IPT
Ansa [40]	2014	Ghana	Comparative research	Facilities	Record review	CPT
Catalani [44]	2014	Kenya	Mixed methods assessment	Providers, Other stake-holders, Facilities	Key informant interviews, Qualitative field notes of site observations, Interviewer-administered survey, In-depth interviews	IPT
Chan [32]	2014	Malawi, Uganda, Zimbabwe	Multi-country comparative study	Facilities	Interviewer administered survey, Record review	CPT
Chang [31]	2015	Uganda	Randomized trial	Patients	Survey-based assessment	CPT
Durovni [45]	2010	Brazil	Preliminary results of phased cluster randomized trial	Patients, Providers, Other stake-holders	Trial dataset record review, Interviews, Focus group discussion	IPT
Faust [46]	2020	Ethiopia, Nigeria, India, Angola, Brazil, China, DRC, Indonesia, Kenya, Lesotho, Liberia, Mozambique, Myanmar, South Africa, Tanzania, Thailand, Zambia, Zimbabwe	Survey	Countries	Survey (via email)	IPT
Gust [47]	2011	Botswana	Sub-study of the Botswana IPT prevention trial	Patients	Interviews, Focus group discussion, Interviewer-administered survey	IPT
Horwood [41]	2010	South Africa	Evaluation/ Cross-sectional descriptive study	Facilities	Record review, Survey-based interviews	CPT
Huerga [48]	2016	Kenya, Swaziland	Two prospective cohort studies	Patients/ Facilities	Record review (cohort study data, clinic registers), Interviews, Observation	IPT
Jacobson [49]	2017	South Africa	Qualitative study	Patients	Semi-structured interviews	IPT
Jarrett [50]	2019	South Africa	Multi-method assessment	Providers, Patients	In-depth interviews, Record review	IPT
Kamuhabwa [14]	2015	Tanzania	Retrospective descriptive study	Facilities, Caregivers, Providers	Record review, Interviewer-administered survey	CPT
Kamuhabwa [35]	2016	Tanzania	Descriptive cross-sectional study	Patients, Providers, Facilities	Record review, Semi-structured interviews, self-administered questionnaires, Focus group discussion, Facility assessment	CPT
Khan [51]	2014	South Africa	Diagnostic performance evaluation	Patients	TB screening questionnaire, Sputum specimens, Chest X-ray, Record review	IPT
Lai [52]	2019	Ethiopia	Cross-sectional study	Providers	Interviewer-administered questionnaires	IPT
Lester [53]	2010	South Africa	Qualitative methods study	Patients, Providers	In-depth interviews, Focus group discussion	IPT
Little [54]	2018	Malawi	Sub-study of the CHEPETSA trial	Patients	Adherence (pills dispensed at each visit), Interviewer-administered questionnaires	IPT
Louwagie [36]	2012	South Africa	Historical cohort study	Facilities	Record review	CPT
Luyirika [39]	2013	Uganda	Retrospective case study	Facilities	Record review	CPT
McRobie [55]	2017	Uganda	Facility-level policy implementation assessment	Facilities, Other stake-holders	Key document review, structured health facility survey, Key informant interviews	IPT
Meribe [56]	2020	Nigeria	Assessment of a provider-focused intervention to increase IPT initiation and completion	Facilities	Review of routinely collected programme data, Health facility quality assessments	IPT
Mindachew [57]	2011	Ethiopia	Analytical cross-sectional study	Patients	Interviewer-administered structured questionnaires, Adherence (self-report)	IPT
Mugomeri [13]	2018	Lesotho	Qualitative study	Providers, Other stake-holders	Semi-structured interviews	CPT, IPT
Mugomeri [58]	2019	Lesotho	Retrospective cohort study	Patients	Record review	IPT
Munseri [59]	2008	Tanzania	Sub-study of the TB vaccine trial	Patients	Record review, Interviewer-administered questionnaires	IPT
Mwambete [34]	2013	Tanzania	Serial clinical and cross-sectional resistance study	Patients	Stool collection for resistance profiling, Adherence (self-report)	CPT
Naikoba [38]	2017	Uganda	Cluster-randomized trial	Providers, Facilities	Practical knowledge/ competence assessment, Record review	CPT
Ngamvithayapong [60]	1997	Thailand	Prospective cohort study	Patients	Adherence (clinic attendance, pill count), Interviews, Focus group discussion	IPT
Okot-Chono [37]	2009	Uganda	Record review, qualitative study	Districts, Patients, Providers, Community members	Record review, Focus group discussion, Key informant interviews, In-depth interviews	CPT, IPT
Okwera [30]	2015	Uganda	Qualitative study	Patients	Focus group discussion	CPT
Reddy [61]	2020	India	Mixed-methods study	Patients, Providers	Review of routinely collected programme data, In-depth interviews	IPT
Rowe [62]	2005	South Africa	Record review, qualitative study	Patients, Providers	Record review, In-depth interviews	IPT
Selehelo [63]	2019	South Africa	Qualitative study	Patients	In-depth interviews	IPT
Sibanda [33]	2015	Zimbabwe	Qualitative study	Caregivers	In-depth interviews	CPT
Szakacs [64]	2006	South Africa	Adherence assessment, Cross-sectional study	Patients	Adherence (urine-metabolite testing, Interviewer-administered questionnaire	IPT
Tram [65]	2019	Uganda	Cross-sectional study (sub-study of SEARCH HIV test and treat trial)	Patients	Record review, Interviewer-administered survey	IPT
Van Ginderdeuren [66]	2019	South Africa	Before-and-after study)	Facilities, Providers	Record review, Structured questionnaire survey	IPT
Wambiya [67]	2018	Kenya	Qualitative study	Providers	In-depth interviews	IPT

Abbreviations: CPT- cotrimoxazole preventive therapy; IPT—isoniazid preventive therapy, Providers—Healthcare providers or healthcare workers

### Risk of bias

We assessed the methodological quality of each study individually and summarized our findings (S4 File). Overall, we identified seven studies with methodological limitations in more than two of the quality criteria evaluated [34, 37, 39, 44, 45, 55, 61]. However, to gain a broader understanding of the barriers and facilitators to PT’s, we did not exclude any study from our analysis. Selective reporting of studies and findings in primary research may have introduced a risk of bias across studies. Similarly, publication bias may have led to a systematic distortion of our understanding of the phenomenon of interest, solely because specific findings are less easily accessible or available.

### Barriers to preventive therapies

#### Patient and community related barriers

**Adverse reactions**, side effects or undesirable reactions were frequently assessed in the included studies [13, 14, 30, 33–35, 45, 47, 52, 57, 59–63, 65–67]. Many of these studies, including two that based their findings on IPT trial data [45, 47], either found that both preventive therapies were generally well-tolerated, that the majority of patients had not experienced any side effects, or both [14, 30, 34, 45, 47, 59, 62, 66]. Co-administration of other drugs (e.g. to treat HIV, TB, hypertension, diabetes, asthma) was common among PLHIV [34, 35, 47, 63] and believed to have contributed to adverse reactions [34, 35, 47, 58]. Pyridoxine shortages and patients’ poor nutritional status were believed to have affected the tolerability of IPT [61]. Side effects influenced patients’ and health providers’ views, attitudes and practices regarding the use and prescription of both therapies [52, 57, 59, 61, 65–67].

**Lacking financial and organisational feasibility** frequently emerged as barrier theme [14, 33, 37, 43, 45, 47, 49, 53, 57, 59–62, 64]. Evidence synthesis revealed that long distance to the clinic [43, 59], lacking transport [14, 43], and with transportation associated costs [43, 45, 47, 49, 53, 62] routinely comprised an obstacle for patients to access therapy and care. Stock-outs at public health facilities led to therapy interruptions among patients who could not afford to buy INH or CTZ at the community pharmacy [14, 33, 37, 64]. Research from Uganda and Brazil suggested that TB screening incurred additional costs for patients [37, 45]. Lack of access to social protection schemes [61] and lack of food security [62] appeared to be a barrier to IPT. Studies from Botswana and South Africa suggested that patients needed to choose between spending a full day waiting at the clinic, dedicating the time to their work [47, 49, 53] or addressing other family members’ needs [49, 62]. Studies from Ethiopia, Botswana, Uganda and Thailand showed that patients did not access treatment or care when they relocated [47], moved far away [43] or were seeking work in other provinces [34, 49, 57, 60].

**Knowledge gaps and misperceptions** regarding both preventive therapies were barriers for patients’ retention in care, therapy adherence and completion [14, 30, 37, 43, 47, 49, 50, 53, 60–63, 66, 67]. Patients in Uganda thought that CTZ is an analgesic drug, that concurrent use of CTZ and ART, or TB treatment is contraindicated, and that CTZ was a treatment for HIV-positive patients [30]. Patients in Thailand thought that INH was prescribed to reduce HIV blood concentration or prevent AIDS-related complications [60]. Patients in South Africa believed that INH would alleviate symptoms, leaving asymptomatic patients unconvinced [49]. Three studies reported that HIV-positive adults were not aware of the desired benefit of IPT in preventing TB [49, 53, 60], and some patients had never heard of IPT [60]. Not knowing the benefit seemed acceptable for some patients [49, 60, 63]. However, most studies suggested that low patient knowledge about IPT is rather harmful for its implementation [43, 47, 49, 60, 62]. Patients in Uganda and South Africa felt better and did not understand the need to return to the clinic or to take IPT, suggesting they lacked understanding of the concept of prevention [43, 50, 62]. Health providers interviewed in Kenya, South Africa and India also acknowledged this problem [50, 61, 67]; some patients refused to take ‘TB medicine’ [INH] without having TB [50, 67]. Others did not value the preventive effect of IPT because of the low perceived risk of TB and lack of symptoms [61]. Health providers reported that information materials were unavailable or inadequate to educate PLHIV, there was a lack of social campaigns and consensus regarding patient education activities [61, 67].

**Patients’ lacking motivation** [14, 43] was identified for both preventive therapies [14, 30, 33, 37, 43, 47, 53, 57, 61–63, 67]. Patients reported they struggled with the need for too many clinic visits, on the one hand, due to a lack of same-day services [37], on the other hand, due to the long waiting time prior to receiving services [37], as well as the long preventive therapy duration [57, 67]. Patients reported the daily pill burden, which was exacerbated through co-medication [53, 63], was an issue [30, 47, 53, 61, 63, 67]. **Forgetfulness** has been frequently reported as a reason for patients’ non-adherence to preventive therapies [34, 47, 57, 64].

**Patients’ HIV denial, religion and competing medicinal approaches** impeded patients acceptability of preventive therapies [33, 35, 43, 47, 60, 62, 65]. Patients’ denial of HIV was a barrier to patients’ retention in care [60], and consequently, their preventive therapy uptake [33]. Non-compliant adult patients interviewed in Uganda and Thailand were not accepting their HIV status and thus, never returned to the clinic, or only months later [43, 60]. Religion and competing medicinal approaches were barriers identified on the sub-Saharan continent. Three studies suggested an influence of religion as an explanation for HIV denial [35, 47, 62]. Interviews revealed that people diagnosed with HIV were encouraged to pray that God will cure their infection, while born-again Christians were advised to stop taking CTZ [35]. Self-reported reasons for non-adherence and loss-to-follow up in the Botswana IPT trial also included religious beliefs [47]. In South Africa, a study participant reported that members of the church could not combine clinic medication with church tea. Similarly, people believed it was prohibited to take clinic medication with traditional medicine [62]. According to Rowe et al. (2005), HIV was perceived as incurable by western medicine, while traditional healers were perceived as able to cure HIV [62]. Among non-completers in Uganda, some patients took traditional medicines ‘to prevent becoming sick with TB’ [65].

**Stigma and fear of rejection or discrimination** remain a barrier for HIV care and consequently for implementing both preventive therapies [33, 35, 37, 43, 47, 49, 59, 60, 62, 64, 65]. Cotrimoxazole and IPT were linked to HIV [35, 59, 62, 65], which although a common infection in the countries included in this review, was frequently associated with stigma and fear of rejection [33, 59, 62] or discrimination [47, 59, 62]. Although mainly reported by women [33, 59], in-depth interviews with HIV-positive mothers in Zimbabwe suggested that men also feared separation from their wives when testing positive [33]. HIV-positive people were laughed at or ridiculed, suggesting a lack of understanding of HIV within the community [33]. Among men, HIV was perceived as emasculating [33]. These HIV-positive patients’ experiences explain their frequently cited unwillingness to disclose their HIV status [33, 35, 49, 59, 60, 62].

**The influence of relatives and friends** seemed an important factor for the implementation of both preventive therapies [30, 33, 35, 43, 47, 59, 61, 62]. Although social and family support seemed to positively influence some patients’ therapy adherence [62], compliance with the prescribed therapy regimen was at threat when patients lacked this support [33, 43, 59, 62, 65]. Some patients felt discouraged by their relatives, who disapproved or sabotaged their treatment adherence [30, 43, 47, 59].

**Socio-demographic, lifestyle and clinical factors** appeared to influence peoples’ use of IPT (i.e. their chances of initiating, receiving, completing or adhering to IPT) [13, 45, 47, 54, 57–59, 61, 65, 67]. Studies suggested that people’s age (being younger) [47, 54, 58, 61, 65], low literacy or educational level [59, 61], their lifestyles [45], including drinking alcohol [47, 61], negatively influenced patients’ use of IPT. With respect to sex, we identified contrasting findings; five studies suggested that being female was positively associated with the use of IPT [47, 54, 58, 60, 61], while one study from Tanzania suggested vice versa [59]. Although there was some consensus that younger age was positively associated with the use of IPT) [47, 54, 58, 61, 65], some subgroups lagged behind. In particular, children (and adolescents) had a lower probability of initiating (or receiving) IPT compared to their adult counterparts [13, 58, 61]. Similarly, pregnant women [13, 47], people aged over 65 [61] and spouses of PLHIV also lagged behind [13, 61]. A higher education level seemed to improve the chances of IPT initiation [61] and completion [59].

**Concern about the efficacy of CPT** resulted from the steady evolution of antibiotic-resistant bacteria. Research from Dar es Salaam, Tanzania, attempted to investigate the incidence of faecal *E*. *coli* resistance to CTZ among HIV patients through in-vitro resistance testing and found considerably high antibacterial resistance. The authors concluded resistance concerns and suggested reconsidering CPT use in Tanzania [34]. Factors that may have contributed to the evolution of CTZ-resistant bacteria include poor adherence to CPT and doctors’ loose prescription practices [30]. The availability of antibiotics without prescription (in pharmacies, parks, or bus stations) may have promoted resistance to CTZ [30, 34]. We did not identify resistance concerns or scepticism among health providers.

#### Health provider related barriers

**Shortage of health providers** working at public health facilities emerged as a barrier for both preventive therapies [13, 35, 37, 42, 44, 45, 50, 55, 61, 63, 66, 67]. However, except from one study that referred to CPT [35], all other studies reported staff shortages as an obstacle for the implementation of IPT. Shortage of health providers was found in a range of countries including South Africa [50, 63, 66], Uganda [37, 55], Kenya [44, 67], Tanzania [35], Nigeria [42], Lesotho [13], Brazil [45], and India [61]. A disproportional provider-patient ratio was reported, suggesting the number of health providers did not allow the additional tasks required to deliver IPT [61, 63, 67]. Studies from Brazil and Kenya, aimed at exploring doctors attitudes to IPT, described doctors’ pressure to see as many patients as possible [44, 45]. Two studies suggested that working conditions (i.e. low salaries, heavy workloads) and missing incentives (i.e. career development prospects) were demotivating for health providers, leading to high staff attrition [37, 55]. Similarly, research from South Africa reported high staff turnover, which seemed to exacerbate the human resources challenges [66].

**Knowledge and training gaps** regarding both preventive therapies were identified among health providers, limiting their ability to make appropriate therapy decisions [13, 35, 37, 38, 41, 43–45, 50, 53, 61, 67]. Regarding CPT, knowledge gaps existed among low and mid-level health providers regarding the co-management of HIV-TB [37, 38], CPT for malaria prevention among HIV-positive pregnant women [35, 41], and CPT for babies born to HIV-positive mothers [41]. Health providers in Tanzania did not have sufficient knowledge about the inclusion criteria (HIV serostatus confirmed, time of initiation, no history of allergic reactions caused by sulfur-containing drugs) and exclusion criteria (skin rashes, Stevens-Johnson syndrome) for CPT [35]. Regarding IPT, knowledge gaps existed among HIV providers with various levels of education, including doctors. Our evidence synthesis suggested that providers were comfortable managing PLHIV [45], but lacked knowledge about TB [43, 45] and TB prevention [13, 44, 45, 50, 53, 61, 67]. Knowledge gaps were related to specific target groups for IPT (i.e. children [13, 61] and pregnant women [61]), IPT duration [50, 67] and the assessment of IPT eligibility [13, 44, 45, 50, 61]. Lacking clarity was also identified about the role of chest x-ray when determining IPT eligibility [44] and regarding the initiation of IPT without placing a TST [50]. Nurses were unsure how IPT duration varied by patient age, TST result, pregnancy and ART status [50], and about the period after which IPT should be repeated [13]. Lack of knowledge and familiarity with IPT among providers was explained by inadequate staff education [13, 37, 44, 61, 67] and minimal follow-up supervision after training [37]. Training modules provided to health providers lacked content on TB-HIV collaborative activities [37] or included limited or no specific training on IPT [67].

**Negative attitudes, concerns and fears** among health providers impeded the delivery of IPT [13, 43, 44, 52, 53, 61, 63, 66, 67]. Our review revealed that health providers often had a negative or sceptical attitude toward IPT, for which we found several explanations [44, 50, 53, 61, 66, 67]. Providers perceived patients’ irregular clinic attendance, non-adherence to IPT and high loss to follow-up as demotivating and discouraging for IPT initiation. Counselling patients about IPT and encouraging them to adhere to preventive therapy was perceived as difficult and time-consuming [66]. In the absence of external oversight (e.g. clinical mentorship), nurses would lose their motivation to prescribe IPT, interviews in South Africa revealed [50]. Health providers’ increasing workload [44, 67], managing IPT among other priorities [67], and the influence of fellow health providers also discouraged them from prescribing IPT [53, 67]. Qualitative research from South Africa aimed to explore patients’ and providers’ attitudes toward IPT. The study found that none of the twenty HIV-patients interviewed had heard of IPT, and doctors were either unaware of the efficacy of IPT in preventing TB or unconvinced about its benefits. Clinic staff and doctors admitted that IPT was not part of routine practice (such as CPT) [53]. Concerns and fears were also related to active TB [13, 43, 63, 66, 67], antibiotic resistance [44, 53, 63, 67] and side effects [13, 52, 67]. In the first place, health providers were concerned about their ability to exclude active TB based on TB symptom screening. Providers who worked in a setting where no TST was available believed that applying the screening algorithm alone was insufficient to rule out TB [66, 67]. Atypical clinical presentations complicated TB screening of patients coinfected with TB-HIV [53], and occasionally, HIV-patients developed TB disease during [43, 63] or after the course of IPT [13, 43, 63]. Bacterial resistance to INH was believed to result from providers’ failure to rule out TB [63], periodic and long-term drug stock-outs [44], patients’ non-adherence to IPT [67] and the use of IPT in settings with a high prevalence of multi-drug and extremely drug-resistant TB [53].

**Provider-patient communication** emerged as a barrier to the implementation of IPT. Our review revealed that patients were often afraid to speak to health providers about IPT, suggesting ineffective provider communication and a lack of trust in the health provider-patient relationship [49, 50, 57]. Jacobson and colleagues (2017) [49] explored patients’ reasons for defaulting from IPT through individual interviews. Interviewees stated they avoided reporting side effects, questioning missing medications, or requesting to re-start IPT after treatment lapses due to their fear of nurses scolding [49]. Research from Ethiopia found that patients lacked advice from doctors, feared side effects and stigma [57]. Similarly, HIV-patients interviewed in South Africa revealed that they felt intimidated during their conversation with nurses; nurses were sometimes rude, which had discouraged them to ask about IPT [50].

#### Clinical information related barriers

**Inaccurate recording and lacking integration of health records** emerged as a barrier for both preventive therapies [13, 35–37, 41, 44, 50, 62, 66]. Although poor recording of clinical information on patients’ records was frequently reported [13, 36, 37, 41, 44, 66], our review revealed that incomplete records did not necessarily imply that services have not been provided. Studies related to CPT [36, 41] and IPT [58, 66] suggest that preventive therapies were delivered more frequently than recorded on patient records. Quantitative research from South Africa, for instance, showed a discrepancy between what has been recorded in IPT registers and what patients had received according to pharmacy records. The authors concluded underreporting of IPT in routine IPT registers [66]. Inaccurate recording of patient information may be explained by weaknesses within the health information system. First, health information systems in high TB/ HIV burden countries were paper-based or relied on a patchwork of paper-based and electronic records [13, 44]. Second, recording tools were described as poorly designed, unpractical [37] and disorganised [50]. Specifically, HIV registers lacked data entry sections for TB-HIV collaborative services; TB information were only recorded on patients’ chronic care cards [37], and patient charts lacked a specific log for IPT registration [50]. Our review revealed risks related to lacking integration of patient data recorded from different service units. Documentation of patient data in separate patient files (e.g. HIV register, patients’ chronic care card, laboratory-, pharmacy record) affected service delivery efficiency by multiplying the time spent for document retrieval, data entry, and in some cases, the provision of services that had already been provided [35, 37, 44]. In the context of TB-HIV co-infection, poor recording can result in the administration of drugs that are contraindicated, risking preventable drug interactions or adverse reactions [35, 62].

**Ineffective monitoring, evaluation and surveillance** emerged as clinical information related barrier [13, 37, 55, 66]. Both concepts, monitoring and evaluation (M&E) and surveillance require collecting data that are analysed and transformed into strategic information that governments and leaders use to make informed decisions. Study data showed that patient data recorded at the health facility level were commonly inaccurate or lost [13, 44]. A study from South Africa showed that IPT indicator data were not comparable due to inconsistent denominators across health facilities. Some facilities routinely collected data to measure IPT uptake among all PLHIV; others used HIV-patients newly enrolled in HIV care as denominator [66]. Others reported a shortage of paper registers [13]. High level key informants in Lesotho suggested that barriers to the implementation of IPT included ineffective health information systems [13]. McRobbie et al. (2017) [55] reported that there were still no regional or national information systems in Uganda that allowed the integration and submission of health facility data. Overall, in these high TB/HIV burden countries, the detection and notification of health events (surveillance) and reporting of routine programme data (M&E) seemed to leak in its foundation [13, 37, 66].

#### Pharmaceutical management related barriers

**Drug stock-outs or shortages** were common for both preventive therapies [13, 14, 32, 33, 35, 37, 44, 49, 50, 55–57, 61, 63, 64, 66, 67]. Except from one study that reported stock-outs in India [61], all other studies reported stock issues in countries located in sub-Saharan Africa. African countries included Kenya [44, 67], South Africa [49, 50, 63, 64, 66], Uganda [37, 55], Tanzania [14, 35], Zimbabwe [33], Nigeria [56], and Lesotho [13]. Inconsistent drug supply, periodic and long-term stock-outs, or shortages of CTZ [13, 14, 32, 33, 35, 37] and INH [13, 44, 49, 50, 55, 56, 61, 63, 64, 66, 67] were obtained from record reviews, reported by patients, health providers, representatives of MoH or partner organizations. It may be noteworthy that stock-outs were also reported for dapsone, an alternative drug to CTZ [13] and pyridoxine, a drug prescribed to prevent peripheral neuropathy caused by INH [13, 61]. Among explanations to why health facilities and pharmacies frequently ran out of CTZ, we found an increasing number of HIV patients [37], inaccurate recording [35], challenges with quantification and forecasting of the prescribed preventive therapy [35, 37]. At the health facility level, we identified the late submission of health facility requisitions [37] or ordering of drug quantities that did not cover the facility demand [35]. Outside the health facility level, we found district-wide stock-outs of INH [50] and scarcity of CTZ at the medical stores’ department or government supplier side [14]. A lack of support from policymakers, programme management [13] or district health departments to adequately supply the lower level units with medicines was also believed to facilitate stock-outs [37, 67]. Ineffective supply chain management [13, 35, 63, 65] and logistical challenges [58] provided alternative explanations for frequent stock-outs. Finally, an uncoordinated implementation of IPT shown by Meribe et al. (2020) [56] also led to long-term INH stock-outs.

**Lack of pharmaceutical personnel** emerged for both preventive therapies [35, 55]. Studies reported that sites in Uganda lacked a dedicated logistics manager [55] and that health facilities lacked pharmacy personnel in Tanzania [35].

**Lack of written instructions** was reported in Tanzania, referring to missing documented strategies for ensuring the availability of CTZ at the health facilities and missing written instructions clarifying how patients should administer medicines at home [35].

#### Service delivery related barriers

**Sub-optimal service delivery** emerged as a critical barrier for the implementation of both preventive therapies. Fourteen studies that reported from a large range of countries with a high burden of TB and HIV [14, 33, 36, 37, 41–43, 45, 49, 50, 55, 61, 63, 67] showed that service delivery was time-consuming, the patient was not put at the centre of care and that health services delivery was inefficient. Service lapses, limited working days, and doctors’ limited working hours have been reported at public health facilities [43, 49]. Studies revealed that patients faced long queues, spending hours waiting before receiving services or their medications [33, 37, 49, 63]. Dispense of IPT and ART in separate lines [49] and the lacking synchronization of IPT with other HIV clinic appointments [42] added to patients’ waiting time and clinic visits. Due to a lack of same-day services, one family had to spend multiple days at the clinic for HIV care [33, 37]. Overall, services delivery routinely tested patients’ willingness and ability to receive HIV care [43]. Among the potential underlying causes of sub-optimal service delivery, the disproportional provider-patient ratio was the most prominent. Human resource constraints presented a critical operational challenge to HIV services delivery [55, 61, 63].

**Inadequate facility infrastructure** emerged as a barrier for both preventive therapies [14, 37, 55]. Health facilities in Tanzania lacked weighing scales to determine infants’ weight for CTZ doses calculation. Thus, providers based the doses on age rather than weight, unable to ensure infants received the correct drug amount [14]. Facilities have been reported as inadequate to ensure patients’ privacy and confidentiality; lacking counselling rooms and inadequate space to implement the HIV policies was reported [37, 55]. Research from Uganda suggested that an outside waiting area for HIV services attendees may raise suspicion among husbands seeing their wives there seated, presuming that they may have contracted HIV [37].

**Poor integration of preventive therapy related services** was another important cross-cutting barrier theme [13, 32, 35, 37, 41, 45, 49, 53, 55, 67] that arose from the segregated delivery of health services in many high TB/HIV burden countries. Studies showed that preventive therapies were frequently not part of the services provided at the health facility [32, 41, 55]. Overall, preventive therapy was poorly integrated with other speciality services [35, 37, 45, 49, 53, 67], often requiring patient referral between health facilities and incurring additional patient costs [37, 67]. However, the inter-clinic referral was reported to be weak [13, 37], added to patients’ costs [67] and challenged service quality [53].

**Patient loss to follow-up** was identified for both preventive therapies [41, 47, 54, 55, 61, 62, 66], referring to patients not linked to care (after HIV diagnosis) and those who stopped attending clinic appointments. Mc Robie and colleagues (2017) [55] argued that recent efforts in Uganda had focused on HIV testing and immediate ART provision without strengthening health facility infrastructure and human resources accordingly to allow the implementation of the complete set of HIV care policies. Consequently, systems failed to maintain patients in care downstream in the care cascade, the authors concluded [55]. Besides difficulties to retain patients in care, engaging those reluctant to present for HIV services (i.e. testing, care and support) from the beginning also remained a challenge for HIV service delivery [62].

**Ruling out TB disease** has been reported as a major barrier to implementing IPT [13, 37, 44–46, 48–53, 61, 63, 66, 67]. Due to the need to rule out TB before therapy initiation and during the course of IPT, delivery of IPT has been perceived as difficult [52]. Since 2011, WHO recommends using a simple algorithm to identify PLHIV unlikely to have active TB, no longer requiring other tests in countries where these are unavailable [2]. Patients who do not report any symptoms of current cough, fever, weight loss, or night sweats are unlikely to have active TB. If no contraindications exist, these patients should be offered preventive therapy [2]. Although symptom-based screening has significantly advanced IPT uptake in low-resource settings [52], we identified challenges associated with this approach. These include doubts about the reliability of TB symptom screening among patients with HIV and TB-HIV, lacking provider capacity to routinely screen TB symptoms, and service delivery inefficiencies. Solely based on TB symptom screening, health providers found it difficult to rule out active TB [52]. Some providers believed that symptom screening alone was insufficient to rule out TB [53, 66, 67]. Opponents of symptom-based screening argued that atypical clinical presentations complicated TB screening of patients coinfected with TB-HIV who frequently suffered from extrapulmonary TB [53]. A diagnostic performance evaluation among HIV patients in South Africa suggested that symptom-based screening was very sensitive and diagnostically useful among patients not on ART, however, less reliable among patients on ART [51]. The high patient load was believed to prevent providers from screening every HIV-patient for IPT eligibility [37, 50]. Research revealed that health providers either lost their motivation to prescribe IPT [50] or continued prescribing IPT without TB symptom screening [63, 66]. Health providers interviewed in India reported that proxy attendance delayed IPT initiation. In the mainly rural study setting, patients often send their caregivers or other attenders to collect their ART refill, making it impossible for providers to assess patients’ eligibility for IPT [61]. However, some countries continued using other recommended tools to determine patients’ eligibility for IPT, i.e. tuberculin skin tests (Brazil, Ethiopia, Indonesia and Thailand) and interferon-gamma release assays (Ethiopia) [46]. Evidence synthesis revealed several limitations for each screening tool. Weaknesses of the tuberculin skin test (TST) were frequently associated with the tuberculin (purified protein derivate) required to carry out the test. We identified shortage of purified protein derivate (PPD), delayed or insufficient supply from the manufacturers [46], challenges of ensuring the quality and correct application of PPD [46, 48], as well as the limited utility of TST in settings where BCG vaccination continued [46]. The need for a second visit for TST reading was a major concern among patients and providers, implying additional time and transport costs [48, 66]. Research from Brazil reported alarmingly long delays involved in the process of TST testing [45]. Proponents of TST argued that providing IPT only for those with latent TB infection (after careful exclusion of active TB) would avoid unnecessarily treating a significant number of patients that do not stand to benefit from IPT [48]. The more recently developed interferon-gamma release assays (IGRAs) address important limitations of TST. Patients are only required to visit the clinic once for blood collection and the result is available within one day. Additionally, prior BCG vaccination does not cause a false-positive IGRA test result. However, due to a lack of budget, lab infrastructure and trained personnel, and insufficient capacity for specimen transport, these relatively expensive tests have hardly been used in TB/HIV high burden countries [46].

**Investigating presumptive TB** is a crucial requirement for all patients with a positive TB screening result [2]. Additionally, drug susceptibility testing should be performed for those who develop TB during the course of IPT [51]. However, evaluating patients with presumptive and confirmed TB appeared challenging [13, 37, 44, 48, 51, 53, 63, 66]. Diagnostic equipment needed to investigate TB, including chest radiography [53], laboratory equipped for the analysis of sputum samples [44] and the identification of resistant TB strains, was scarce [51]. Most health facilities could not diagnose TB on-site [51, 53]. Although the more accurate GeneXpert^®^ test allows rapid diagnosis and identification of patients with drug-resistant TB, this expensive molecular test technology was often only available at centralised sites, reserved for a subset of complex cases [13, 44]. Besides, our review revealed knowledge gaps among health providers related to their diagnostic competences [37, 44] and co-management of TB-HIV [37]. In Kenya, doctors specialised in HIV reported they were unsure how to read a chest radiograph [44]. Providers in Uganda reported struggling with the assessment of smear-negative and extra-pulmonary TB [37]. Two studies reported that only a minority of PLHIV with self-reported symptoms were investigated for TB [48, 66], causing TB treatment delays [63] or resulting in too many patients denied for IPT [48].

#### Health system financing related barriers

**Health system funding difficulties** were identified for both preventive therapies [13, 14, 46, 55]. Many high TB/HIV burden countries historically depended on funding for HIV and TB from international donors and governments. Many will continue to require donor backing to improve the provision of a comprehensive care package to PLHIV [55]. Lacking funds to purchase essential medicines (as which CTZ and INH are classified) has been reported in a Tanzanian study setting [14]. Qualitative research from Lesotho found that insufficient health system funding was a challenge for implementing IPT [13]. A recent email survey was carried out to identify challenges experienced in countries with a high burden of TB regarding the implementation of latent TB infection policies. Due to financial barriers, guidelines were not fully implemented, respondents from six high TB/HIV burden countries reported [46].

**Vertical funding** emerged as a barrier for both preventive therapies [13, 37, 55], referring to a stand-alone programme approach (e.g. HIV programme, TB programme) used in many resource-constrained countries for health system funding, typically associated with vertical governance and health service delivery. Both preventive therapies require some degree of collaboration between disease programmes (i.e. HIV, TB, PMTCT, child health) to ensure their successful implementation. Lacking funds for implementing TB/HIV collaborative activities in Uganda was reported on a national TB/HIV programme level and district level [37, 55]. Policymakers noted international donor dependency of the HIV programme [55] and misalignment of goals between the government and donors, suggesting lacking donor support for health systems strengthening and prevention activities [55]. Donors were considered to focus more on the provision of ART [55], while funding of human resources primarily rested with the individual Ministry of Health [13, 55].

#### Leadership & governance related barriers

**Issues regarding policies and guidelines** emerged for both preventive therapies [13, 14, 37, 46, 50, 53, 66, 67]; however, they were most commonly reported for IPT. A recent survey [46] suggested that guidelines on latent TB infection (LTBI) did not exist in several high TB/HIV high burden countries (Angola, China, DRC, India, Indonesia, Kenya, Myanmar) [46]. Several studies revealed that preventive therapy guidelines were not always accessible [14, 37] and understandable for health providers [13, 14, 37, 50, 67]. In Kenya, South Africa and Lesotho, national guidelines for IPT were described as unclear [13, 67], ambiguous, confusing, and incomplete [50]. In summary, guidelines lacked clarity about eligibility criteria, the duration of IPT, how to decide whether a patient had active or latent TB [67], and the duration after which IPT should be repeated [13]. Lacking clarity might have contributed to health providers’ low fidelity to the IPT guideline in South Africa [53, 66].

**Lacking leadership and coordination** emerged for both preventive therapies [13, 37, 41, 45, 49, 50, 67]. Weak leadership and coordination between disease programmes at the strategic and operational level have represented a major challenge for TB/HIV collaborative activities [37, 45, 67]. Studies showed that TB and HIV services’ responsibilities remained fragmented [45, 49], lacking clarity concerning roles and responsibilities for collaborative service delivery at the health facility level [37, 41]. Lack of leadership was reported as a barrier to scaling up IPT for PLHIV in Lesotho [13].

**Lacking top-down policy support and management issues** emerged as barriers for both preventive therapies [37, 49, 50, 67]. On an institutional and policy level, lack of commitment, support and oversight hindered the implementation of IPT policies and collaborative TB/HIV activities. Providers interviewed in Kenya felt demotivated by the limited commitment at the policy level in ensuring effective implementation and streamlining of the IPT programme [67]. After investigating patients’ reasons for defaulting IPT, Jacobson et al. (2017) [49] concluded that long queues, drug stock-outs, and a lack of service integration require attention on an institutional and policy level. At the health facility level, ‘no-one was overseeing’ the implementation of TB/HIV collaborative services, participants of a study conducted in Uganda reported [37]. Inadequate joint supervision or external oversight may have led to providers’ poor performance [37] or lack of motivation to prescribe IPT [50].

**Inadequate planning** was identified as a barrier to the implementation of IPT and TB-HIV collaborative activities [13, 37]. Key informant interviews suggested inadequate national planning as an important barrier to IPT implementation in Lesotho; IPT was affected by lack of foresight at the planning stage and poor capacity to solve problems that arise [13]. Multimethod research from Uganda revealed that out of five districts implementing TB-HIV collaborative activities, only one had incorporated collaborative activities into its district work plan. The remaining four districts had separate HIV and TB plans without TB-HIV joint activities. Patients and communities were not involved in the planning process [37].

**Lacking stakeholder engagement** emerged as a barrier theme for both preventive therapies, suggesting that community consultation and engagement of implementers and the population targeted for preventive therapies were insufficient [13, 37, 67]. According to research from Uganda, community-related activities were planned without community involvement. Additionally, the study reported a lack of information flow from health facilities to the communities concerning the TB and TB-HIV services available [37]. Limited engagement of health providers in the development of the IPT guideline may explain their low acceptability of IPT. Health providers interviewed in selected HIV clinics in Kenya reported they were told: “Here are the guidelines to be followed!”. Providers felt they should have been involved in the process of guideline development [67]. Lacking engagement of the people responsible for implementing IPT (i.e. health providers) was reported to inhibit IPT uptake in Lesotho [13].

#### Metasummary

**Fig 2:**
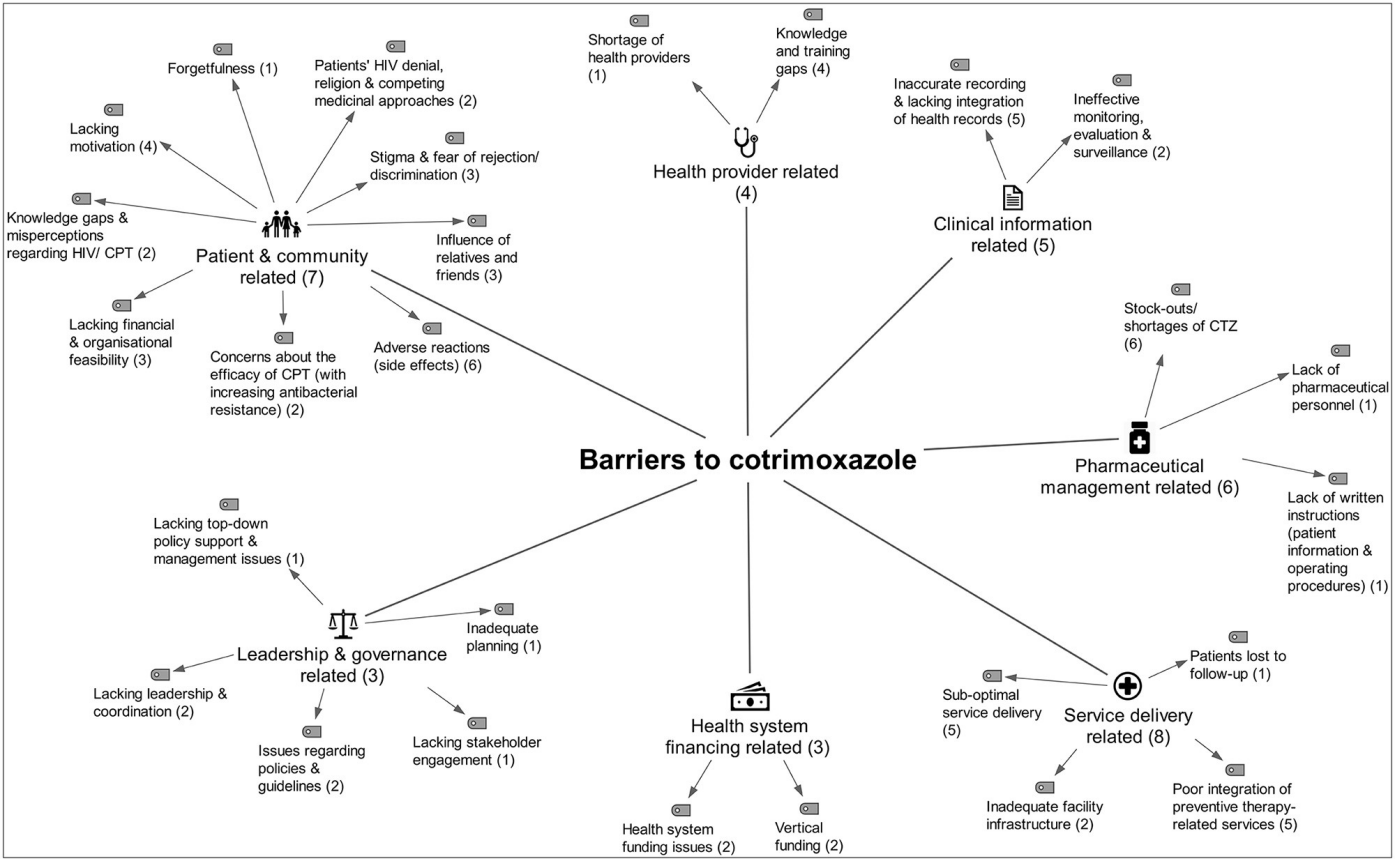
Metasummary: Barriers to cotrimoxazole preventive therapy for people living with HIV. The graphic presents all main barrier themes identified in countries with a high burden of tuberculosis and HIV, assigned to the health system component from which the barrier theme arose, considering a seven component health system framework (modification of the WHO Framework for Action, 2010). Barrier themes identified in more than or equal to ten papers are presented with an exclamation mark. Numbers in brackets display the number of supporting papers. Total number of peer-reviewed papers included with barriers to cotrimoxazole in this review (N = 11).

**Fig 3:**
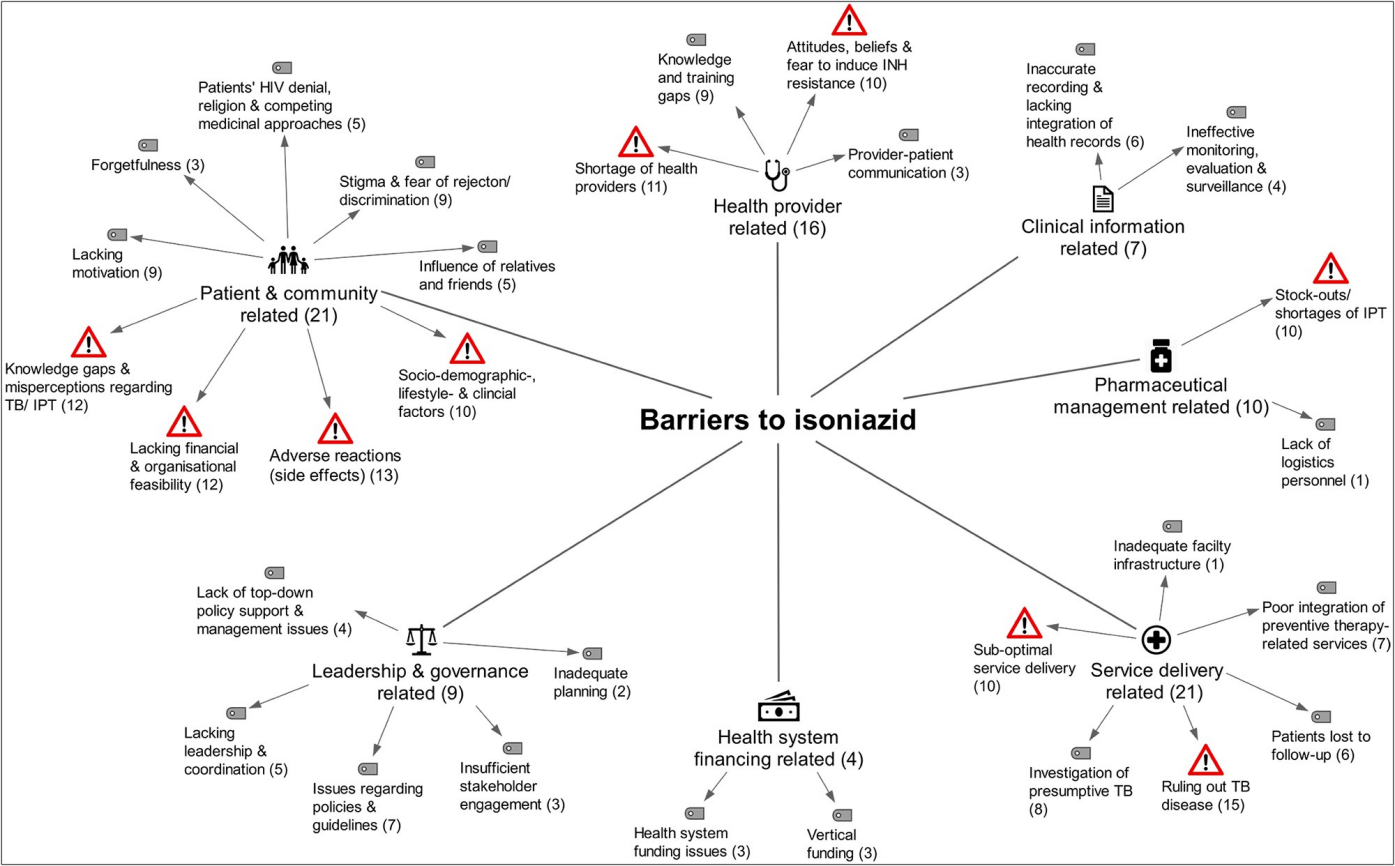
Metasummary: Barriers to isoniazid preventive therapy for people living with HIV. The graphic presents all main barrier themes identified in countries with a high burden of tuberculosis and HIV, assigned to the health system component from which the barrier theme arose, considering a seven component health system framework (modification of the WHO Framework for Action, 2010). Barrier themes identified in more than or equal to ten papers are presented with an exclamation mark. Numbers in brackets display the number of supporting papers. Total number of peer-reviewed papers included with barriers to isoniazid in this review (N = 28).

Overall, thirty-two barrier themes emerged from this review, of which the majority were cross-cutting barrier themes (n = 25). We also identified seven intervention-specific themes. Barrier themes specific to CPT (n = 2) were ‘concerns about the efficacy of CPT’ in areas of high bacterial resistance [30, 34] and a ‘lack of written instructions’ [35]. Barrier themes specifically identified for IPT (n = 5) include ‘patients’ socio-demographic, lifestyle and clinical factors’ [13, 45, 47, 54, 58–61, 65, 67], ‘providers’ attitudes, beliefs & fears to induce INH resistance’ [13, 43, 44, 50, 52, 53, 61, 63, 66, 67] and ‘provider-patient communication’ [49, 50, 57]. Additionally, we identified issues with ‘ruling out TB disease’ [13, 37, 44–46, 48–53, 61, 63, 66, 67] and ‘the investigation of presumptive TB’ as intervention-specific barriers for IPT. We found that barriers to both preventive therapies were most frequently related to ‘health service delivery’ and ‘patients & their community’. For IPT, health provider related barriers played an additional important role, third most frequently referred to. Barriers to CPT were third most frequently related to ‘pharmaceutical management’. ‘Financing’ was among the health system components least frequently associated with barriers to preventive therapies. We mapped all identified barrier themes within each of the seven health system components, together with the number of papers that contributed information to each barrier theme. The resulting graphics allow visual comparison of barrier themes identified for CPT (Fig 2) and IPT (Fig 3).

### Facilitators for the implementation of preventive therapies

**Fig 4:**
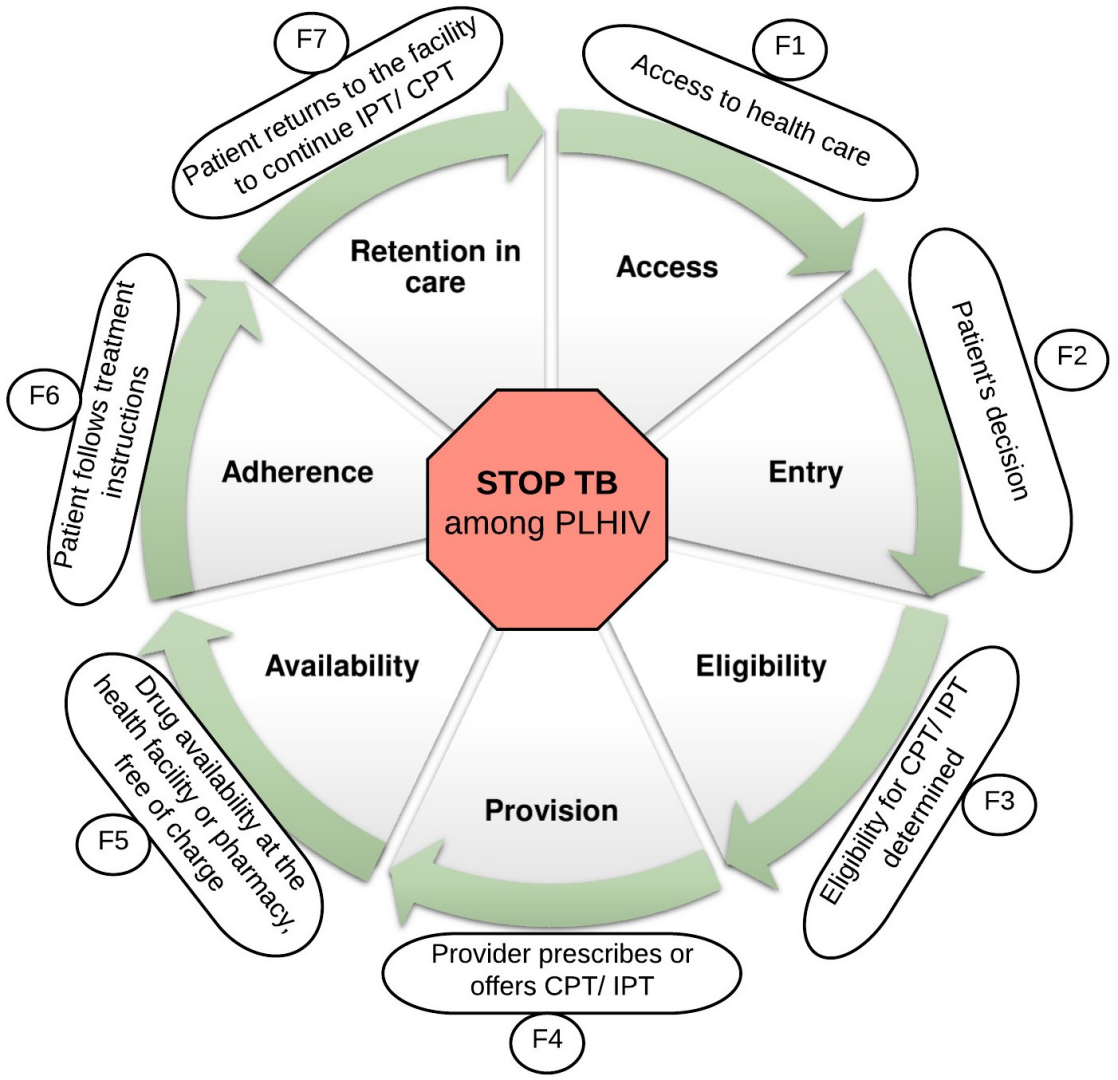
The preventive therapy cascade. Abbreviations: TB—tuberculosis; HIV—human immunodeficiency virus; PLHIV- people living with HIV; IPT- isoniazid preventive therapy; CPT—cotrimoxazole preventive therapy.

We presented our findings according to the preventive therapy cascade—a coding framework we developed a priori (Fig 4).

The preventive therapy cascade outlines the steps that PLHIV repeatedly go through, from having access to appropriate healthcare to completing preventive therapy. We developed this conceptual framework to promote stakeholder discussion and listed the identified facilitators for the implementation of both preventive therapies accordingly. This approach allows (1) selecting steps within the cascade that require attention in a specific context (Fig 4) and (2) rapidly screening potential strategies that address these ‘weak spots’ within the cascade (S5 File).

## Discussion

In high TB/HIV burden countries, both preventive therapies in the centre of this review (i.e. CPT, IPT) are key interventions for PLHIV that can save lives if administered together with ART, or even on their own [6, 9]. However, governments of high TB/HIV burden countries face major implementation challenges that limit the effectiveness of both preventive therapies [13, 14]. We explored and compared both preventive therapies with respect to similarities and differences of barriers identified across high TB/HIV burden countries published in peer-reviewed literature. Additionally, we explored and summarised strategies (facilitators) with the potential to tackle the identified barriers.

### Similarities and differences in barriers

The detailed barrier description presented in this review offered various explanations to why the implementation of both preventive therapies has been so challenging in the concerned countries. Careful analysis of the extracted barrier information showed that many barriers were very similar for both preventive therapies. The majority of barrier themes (25/32) that emerged from this review were identical for CPT and IPT, thus not intervention-specific. This leads to the question: "What are the underlying patterns of these cross-cutting barrier themes?" Considering that cross-cutting barrier themes were scattered across all building blocks of the health system and rather generic in nature indicates that systematic weaknesses within the health system may be an important underlying reason for implementation struggles. All barrier reporting studies, except one [51], reported such cross-cutting barriers, which at least hints that many high TB/HIV burden countries’ health systems were weak and unable to meet the basic requirements of a well-functioning health system [26]. Fragile public health systems could be explained by the fact that more than three-quarters of the countries with a high burden of TB and HIV are low- and lower-middle-income countries (as per 2021 World Bank’s classification) [68]. Many of these countries have severely constrained health budgets and historically depend on foreign assistance [69]. Following two decades of increasing domestic and generous international funding that has been mainly channelled into vertical health programs, impressive gains have been achieved in reducing AIDS-related deaths [26]. However, health systems strengthening is not characteristic for vertical funding and healthcare organisation. A major concern is that impressive gains of the past have been neither universal nor sustainable [26]. We speculate that the sustainability of the HIV response and health facility-based delivery of both preventive therapies will become more challenging over the next years. Besides the consequences of the current SARS-CoV-2 pandemic, the dilemma is that funding from international donors is flatlining [69]. At the same time, an increasing number of HIV patients require life-long medical care [70] to be delivered by vertical programs that have long reached the limit of their effectiveness. At this stage of understanding, we believe it is quite possible that health system constraints identified for CPT and IPT, similarly hinder the implementation of other health facility-based interventions in high TB/HIV burden countries. Therefore, in addition to intervention-specific barriers, context-specific health system constraints should be considered to move towards a more feasible implementation strategy.

Like for CPT, barriers identified for IPT were most frequently classified as ’service delivery-’ or ’patient & community related’. Typically, ’service delivery related barriers’ were interrelated with constraints identified across other health system components. For instance, with inaccurate clinical information, insufficient health providers and frequent drug stock-outs, efficient and reliable delivery of preventive therapy is unrealistic. These interrelations highlight that service delivery strongly depends on a well-functioning interplay of the health system components represented in the original WHO’s Framework for action [26]. Among all ’patient and community-related barriers’, we would like to draw special attention to ’lacking financial and organisational feasibility’. This barrier theme was supported by more than one third of all studies included in this review [12, 15, 16, 22, 23, 25, 28, 31, 40–45], suggesting that in resource-constrained countries and in the context of poverty, free provision of health facility-based services may not be sufficient to ensure equitable access to healthcare. Overall, our review suggests that implementing IPT has been more troublesome than CPT, supported by more barrier themes and evidence reporting barriers to IPT. This is consistent with the cross-sectional study carried out by WHO HIV/AIDS programme officers in 2007 [71], which showed that less progress had been made in implementing IPT when compared to CPT at the time. Thus, more research focus may have been on IPT since. Nevertheless, we argue that there is also more complexity involved in the eligibility assessment and provision of IPT compared to the more straightforward provision of CPT.

Considering challenges specific to implementing IPT, ’health provider related barriers’ appeared to play an important role. Our review revealed that having to rule out active TB disease was an important issue for the implementation of IPT. Local policies and guidelines were described as unclear, so that health providers lacked knowledge and confidence in determining who is eligible for IPT [13, 67]. Health providers appeared to be overwhelmed with this additional task of routinely assessing and documenting patients’ eligibility [37, 50]. While health providers attitudes towards CPT seemed neutral or positive, providers’ attitudes and beliefs toward IPT were partially negative [13, 43, 44, 52, 53, 61, 63, 66, 67]. One health provider concern was that extrapulmonary TB, common among PLHIV, may go undetected when screening for pulmonary symptoms [53]. Some health providers appeared to be unconvinced whether the benefits of IPT outweigh the risks of side effects [13, 52, 67] or of promoting INH resistance [44, 53, 67]. Overall, the use of preventive therapies has often been controversial, with concerns about drug resistance forming one major barrier to policy development and implementation [72]. However, it was somewhat surprising that studies reported health providers’ concern about bacterial resistance for IPT [44, 53, 67], but not for CPT. Notably, many high TB/HIV burden countries witness a high burden of MDR-TB [3]. Instead of INH monoresistance, health providers’ genuine concern may thus lay in promoting MDR-TB among patients with HIV [53]. Another surprising barrier theme identified specifically for IPT was provider-patient communication [50, 51, 58], which could indicate that communication needs are greater for IPT or that patient expectations are changing [73]. Another important factor for the implementation of IPT is that, in contrast to CPT, IPT requires greater coordination between HIV and TB programs at all levels. Many high TB/HIV burden countries have implemented symptom-based screening to determine patients eligible for IPT [46], which is believed to have significantly advanced IPT uptake in low-resource settings [52]. On the one hand, symptom-based screening enabled HIV services units to take greater responsibility for implementing IPT. On the other hand, findings of the studies included in this review suggest that TB services’ capacity and linkage was too weak to follow-up patients with presumptive TB and to ensure immediate TB treatment for patients with confirmed TB [13, 37, 44, 48, 51, 53, 63, 66]. This leads to another challenge that is particular for IPT; it requires capacity for the co-management of TB. Considering challenges specific to the implementation of CPT, we found that bacterial resistance was a concern raised by the authors of a study included in our review [34]. Mwambete and Kamuhabwa (2016) questioned the efficacy of CTZ in areas of high bacterial resistance as a result of their research findings. Based on the disk diffusion method, their study identified an overall high resistance of isolated enteric *E*. *coli* among HIV patients in Tanzania [34]. Although antimicrobial resistance is a topic of utmost urgency, there are reasons to doubt the urgent need to reconsider the use of CPT in high TB/HIV burden countries. On the one hand, the in-vitro assay applied in the study [34] is no longer the gold standard for antimicrobial susceptibility testing because it often disqualifies antibiotics that are, in fact, effective in-vivo [74, 75]. On the other hand, several studies have shown significant reductions in morbidity and mortality among PLHIV on CPT, despite being carried out in settings with high bacterial resistance. Therefore, others have argued that in-vitro resistance testing undermines the prophylactic ability of CTZ [76]. Finally, one study included in our review suggested a lack of written instructions for patients [35]. Although we revealed several knowledge gaps and misperceptions among patients in our review that could be addressed in written instructions, the provision of patient information leaflets, in general, is not yet standard practice in many low- and middle- income countries [77]. One cited explanation for the under-utilisation of information leaflets at public pharmacies is that they are poorly understood [78]. Poor understanding may be more pronounced among populations with low literacy, which may have led to low prioritisation of creating such written information in the concerned countries. However, in combination with pictograms, basic written information has shown valuable for educating patients about CPT [77]. Nevertheless, such patient leaflets are still not available in all countries, reemphasising the importance of effective provider-patient communication during direct patient contact.

### Facilitators to preventive therapies

As barriers vary between and within high TB/HIV burden countries, there is not one strategy for improving the implementation of either or both preventive therapies that fits all settings. We, therefore, encourage redesigning an implementation strategy that addresses barriers identified in a specific context. The awareness of implementation challenges and political willingness to tackle these issues are critical first steps for continuous improvement. Routinely collected indicators, district-level evaluations, dialogues with implementers or local research findings can help identify barriers on a national level or in a specific setting. Our review identified only a few intervention-specific barriers and highlighted that cross-cutting barriers often affect the implementation of both preventive therapies and potentially other interventions in high TB/HIV burden countries. Acknowledging that strategies are needed to target systematic weaknesses can be daunting. However, integrating the concept of health systems strengthening into an implementation strategy provides new opportunities for policymakers and health systems.

Health systems strengthening may still appear unrealistic in low-income countries where primary care facilities struggle to provide basic health services and essential medicines. However, the decision for ‘organisational transformation’ is not based on the capacity of an individual primary care facility but rather the potential behind removing cross-programmatic duplications and leveraging the existing resources. Adapting the implementation strategy of a high-impact multi-programmatic intervention, such as IPT, screams for a more sustainable health system transformation. We acknowledge that health systems strengthening is ambitious to achieve holistically. However, targeted strengthening of a selected health system component, such as up-skilling leadership and governance, reinforcing health providers’ capacity and knowledge, strengthening supply chains or health information systems comprise examples frequently identified in this review with the potential to yield benefits across both preventive therapies and a wide range of other health interventions. Cost-effectiveness analysis can assist in establishing the optimal balance between systems strengthening and intervention-specific approaches [79]. Vertical programmes of the past were popular for their ‘quantifying of lives saved’ approach and short term objectives that promised quick and measurable results [80]. However, disease-specific programmes and initiatives were criticised for their costly parallel structures, less sustained impact, and for negatively affecting non-HIV care [80, 81]. In high TB/HIV burden countries that depend on external funds to finance their HIV response, vertical programming was associated with donor-driven policy evolution and investment decisions that were not always aligned with national priorities [55]. ‘Pushing’ global recommendations without considering context-specific constraints undermines the competence of local policymakers, can create tension between donors and recipient countries’ governments and weaken local policy ownership. Many of these issues have been recognised internationally, and the global health systems strengthening movement has finally gained momentum [82, 83]. Major HIV funding bodies (i.e. USAID, Global Fund) have begun promoting the ‘de-verticalisation’ of fragmented programming [82, 84]. The U.S. Agency for International Development (USAID) and the Global Fund to fight AIDS, TB and Malaria (Global Fund) have incorporated systems strengthening objectives into their strategy [82, 84] and updated their policies to include funding agreements for health systems strengthening that allow direct disbursement of funds to the recipient country’s state budget [80, 85]. Dialogues between donors and local system stakeholders (e.g. USAID’s Government to Government (G2G) assistance, Global Fund’s country coordinating mechanism (CCM)) support alignment of policies with country priorities [80, 85], encouraging mutually beneficial decisions and better relationships between donors and recipient countries. Additionally, the new implementation science culture encourages consideration of national, regional and local lessons learnt [45, 62, 86].

### Strengths and limitations

The main strength of this review is the in-depth understanding that this review provides about the challenges associated with the implementation of CPT and IPT in countries with a high burden of TB and HIV. To explore the existing body of evidence holistically, (1) we applied a comprehensive search strategy, (2) included all stakeholders involved in the implementation process as study population, (3) integrated research with different study designs. The integration of studies with different study designs further enhanced the richness of our findings. Mixed methods systematic reviews have gained increasing scientific attention in the areas of public health and complex interventions, where decision-makers typically require consideration of different types of data and information (e.g. accessibility, feasibility, patient values and preferences) [17]. The application of metasummary to compare interventions with respect to similarities and differences of barriers employed in our review is innovative.

Although our search strategy was systematic and rigorous, it only included peer-reviewed publications reported in the English language. This is unlikely to be a major limitation considering that twenty-three high burden countries were represented in this review. However, this may explain why most studies included in this review were carried out in South Africa, Uganda and Tanzania. Similarly, the study populations represented in our review were represented heterogeneously. The views of caregivers, community members and other stakeholders involved in the implementation process were less frequently described than the perspectives of patients and health providers, limiting the extent to which our findings holistically represent the phenomena of interest. Non-availability or non-dissemination of studies may have limited the completeness of our study findings [29]. Studies on the subject of this review may be less likely to be available in countries with limited political interest to implement the concerned preventive therapies. In high TB/HIV burden countries with little research capacity, available study findings may be more likely to be published in local journals not indexed in major databases.

### Conclusion

For policymakers who encounter challenges with the implementation of either or both preventive therapies, this review offers a list of strategies for improving the implementation of both preventive therapies. Based on evidence from high TB/HIV burden countries, this review includes directions for improving the delivery of IPT. For researchers with limited working experience in high TB/HIV burden countries, this review can provide useful insights regarding to which barriers may arise at different levels of the health system. The barrier description provided in this review highlights the complexity of social interactions involved in the delivery of preventive therapies. Overall, our study showed that until today, many high TB/HIV burden countries’ health systems are not prepared to ensure appropriate public healthcare for the ever-increasing number of patients in need of HIV services. From this standpoint, health system strengthening is imperative for the sustainable delivery of both preventive therapies for PLHIV, but particularly for IPT. Based on our findings, we suggest considering two important aspects to implementation. First, we recommend the early engagement of stakeholders when shaping an implementation strategy. Involving patients and health providers in the process of policy development and planning may increase awareness and understanding about the intervention and help ensure its acceptability. If appropriate, additional stakeholders (e.g. church leaders, traditional healers) may be involved. Second, during the adaptation of an implementation strategy, we urge that attention is paid to the fact that many of the countries with the highest burden of TB and HIV to date are also among the most resource-constrained countries. Thus, novel strategies for the implementation of preventive therapies may appear encouraging at first. Still, in high TB/HIV burden settings, they are often far from feasible or sustainable on a big scale. Instead, we encourage innovative approaches that consider the resources available at the health facility level and invest in strengthening the existing capacities. Future research is warranted to test and evaluate alternative service delivery approaches that reduce the financial and organisational burden that patients face during the course of preventive therapy. Replacing INH with one of the shorter treatment regimens recommended by WHO for the prevention of TB [2] and the community-based delivery of selected activities related to the provision of PT’s may comprise two options with the additional benefit of reducing the patient load at the overburdened health facilities.

## Supporting information

S1 File PICo framework (modified PICO). (PDF)

S2 File PRISMA checklist. (PDF)

S3 File Database searches and results. (PDF)

S4 File Assessment of methodological strengths and limitations. (PDF)

S5 File List of facilitators for the implementation of CPT and IPT. (PDF)
