# Supplementary material for: Mixed methods systematic review and metasummary about barriers and facilitators for the implementation of cotrimoxazole and isoniazid—Preventive therapies for people living with HIV
Source: PLoS One. 2022 Mar 1;17(3):e0251612. doi: 10.1371/journal.pone.0251612 (PMC8887777; doi:10.1371/journal.pone.0251612)
Supplement: S3 File — (PDF) [file pone.0251612.s003.pdf]

Additional file 3. Database searches and results.

Database	Detailed Search expression	Results 8th of February 2018	Results 4th of September 2020
MEDLINE (via Pubmed)	#1 Cotrimoxazol* OR Co-trimoxazol* OR "Trimethoprim, Sulfamethoxazole Drug Combination"[Mesh].<br>#2 Isoniazid OR "Isoniazid"[Mesh].<br>#3 barrier* OR facilitat* OR challeng*.<br>#4 implementation OR provision<br>**(#1 OR #2) AND #3 AND #4**<br><br>(Cotrimoxazol* OR Co-trimoxazol* OR "Trimethoprim, Sulfamethoxazole Drug Combination"[Mesh] OR Isoniazid OR "Isoniazid"[Mesh]) AND (barrier* OR facilitat* OR challeng*) AND (implementation OR provision)	n= 95	n = 126
Web of Science (all databases)	#1 Cotrimoxazol* OR Co-trimoxazol* OR "Trimethoprim, Sulfamethoxazole Drug Combination".<br>#2 Isoniazid.<br>#3 barrier* OR facilitat* OR challeng*.<br>#4 implementation OR provision.<br>**#1 AND #2 AND #3 AND #4**<br><br>TOPIC: (Cotrimoxazol* OR Co-trimoxazol* OR "Trimethoprim, Sulfamethoxazole Drug Combination" OR Isoniazid) AND TOPIC: (barrier* OR facilitat* OR challeng*) AND TOPIC: (implementation OR provision)<br>Indexes=SCI-EXPANDED, SSCI, A&HCI, CPCI-S, CPCI-SSH, ESCI, CCR-EXPANDED, IC Timespan=All years	n= 143	n = 156
Scopus	#1 Cotrimoxazol* OR Co-trimoxazol* OR "Trimethoprim, Sulfamethoxazole Drug Combination".<br>#2 Isoniazid.<br>#3 barrier* OR facilitat* OR challeng*.<br>#4 implementation OR provision.<br>**(#1 OR #2) AND #3 AND #4**<br><br>(TITLE-ABS-KEY ((cotrimoxazol* OR co-trimoxazol* OR "Trimethoprim,Sulfamethoxazole Drug Combination" OR isoniazid)) AND TITLE-ABS-KEY (barrier* OR facilitat* OR challeng*) AND TITLE-ABS-KEY (implementation OR provision)) AND DOCTYPE (ar)	n = 146	n = 200
