# Supplementary material for: Mixed methods systematic review and metasummary about barriers and facilitators for the implementation of cotrimoxazole and isoniazid—Preventive therapies for people living with HIV
Source: PLoS One. 2022 Mar 1;17(3):e0251612. doi: 10.1371/journal.pone.0251612 (PMC8887777; doi:10.1371/journal.pone.0251612)
Supplement: S4 File — (PDF) [file pone.0251612.s004.pdf]

Additional file 4. Assessment of methodological strengths and limitations.

Category of study designs: QUAL

First author [citation]	Methodological quality criteria	Jacobson [1]	Lester [2]	Mugomeri [3]	Okwera [4]	Selehelo [5]	Sibanda [6]	Wambiya [7]
Screening questions (for all types of study designs)	S1	Y	Y	Y	Y	Y	Y	Y
	S2	Y	Y	Y	Y	Y	Y	Y
1. Qualitative (ethnography, phenomenology, narrative research, grounded theory, case study, qualitative description)	1.1.	Y	U	Y	U	Y	U	Y
	1.2.	Y	Y	U	Y	Y	Y	Y
	1.3.	Y	Y	Y	U	Y	Y	Y
	1.4.	Y	Y	Y	Y	Y	Y	Y
	1.5.	Y	Y	Y	Y	Y	Y	Y
2. Quantitative (randomised controlled clinical trial, cluster or individual, randomised cross-over study)	2.1.							
	2.2.							
	2.3.							
	2.4.							
	2.5.							
3. Quantitative (non-randomised studies, e.g. non-randomised controlled trials, cohort study, case-control study, cross-sectional analytical study)	3.1.							
	3.2.							
	3.3.							
	3.4.							
	3.5.							
4. Quantitative (descriptive, e.g. incidence or prevalence study without a comparison group, survey, case series, case report)	4.1.							
	4.2.							
	4.3.							
	4.4.							
	4.5.							
5. Mixed methods (convergent design, sequential explanatory design, sequential exploratory design)	5.1.							
	5.2.							
	5.3.							
	5.4.							
	5.5.							

Category of study designs: QUAN

First author [citation]	Methodological quality criteria	Chang [8]	Naikoba [9]	Adepoju [10]	Ansa [11]	Khan [12]	Little [13]	Louwagie [14]	Mindachew [15]	Mugomeri [16]	Munseri [17]	Ngamvithayapong [18]	Szakacs [19]	Tram [20]	Van Ginderdeuren [21]	Aisu [22]	Chan [23]	Faust [24]	Horwood [25]	Kamuhabwa [26]	Lai [27]	Meribe [28]	Mwambete [29]
Screening questions (for all types of study designs)	S1	U	Y	Y	Y	Y	Y	Y	Y	U	Y	Y	Y	Y	Y	Y	Y	Y	Y	Y	Y	Y	U
	S2	Y	Y	Y	Y	Y	Y	Y	Y	Y	Y	Y	Y	Y	Y	Y	U	Y	Y	Y	Y	Y	Y
1. Qualitative (ethnography, phenomenology, narrative research, grounded theory, case study, qualitative description)	1.1.																						
	1.2.																						
	1.3.																						
	1.4.																						
	1.5.																						
2. Quantitative (randomised controlled clinical trial, cluster or individual, randomised cross-over study)	2.1.	Y	Y																				
	2.2.	Y	Y																				
	2.3.	Y	Y																				
	2.4.	N	Y																				
	2.5.	Y	Y																				
3. Quantitative (non-randomised studies, e.g. non-randomised controlled trials, cohort study, case-control study, cross-sectional analytical study)	3.1.			Y	Y	Y	N	Y	Y	Y	N	Y	N	Y	Y								
	3.2.			Y	Y	Y	Y	Y	Y	Y	Y	Y	Y	Y	Y								
	3.3.			N	Y	Y	Y	Y	Y	Y	Y	Y	Y	Y	Y								
	3.4.			Y	N	Y	Y	Y	U	Y	Y	Y	Y	Y	Y								
	3.5.			N	U	Y	Y	Y	U	U	U	Y	Y	Y	U								
4. Quantitative (descriptive, e.g. incidence or prevalence study without a comparison group, survey, case series, case report)	4.1.															Y	N	Y	Y	Y	Y	Y	Y
	4.2.															Y	Y	Y	Y	Y	Y	N	U
	4.3.															Y	Y	U	Y	Y	Y	Y	N
	4.4.															/	Y	N	U	U	Y	U	U
	4.5.															/	/	/	Y	/	Y	/	Y
5. Mixed methods (convergent design, sequential explanatory design, sequential exploratory design)	5.1.																						
	5.2.																						
	5.3.																						
	5.4.																						
	5.5.																						

Category of study designs: MULTI and MIX

First author [citation]	Methodological quality criteria	Durovni [30] (MULTI)	Gust [31] (MULTI)	Rowe [32] (MULTI)	Huerga [33] (MULTI)	Kamuhabwa [34] (MULTI)	Jarrett [35] (MULTI)	Luyirika [36] (MULTI)	McRobie [37] (MULTI)	Okot-Chono [38] (MULTI)	Catalani [39] (MIX)	Reddy [40] (MIX)
Screening questions (for all types of study designs)	S1	Y	Y	Y	Y	Y	Y	Y	U	U	U	Y
	S2	Y	Y	Y	Y	Y	Y	U	Y	Y	Y	Y
1. Qualitative (ethnography, phenomenology, narrative research, grounded theory, case study, qualitative description)	1.1.	U	Y	U		U	U	U	U	U	Y	U
	1.2.	U	Y	Y		Y	Y	U	Y	Y	Y	Y
	1.3.	U	Y	Y		Y	Y	N	Y	Y	Y	Y
	1.4.	N	Y	Y		Y	Y	N	Y	N	Y	Y
	1.5.	U	Y	Y		Y	Y	N	Y	U	Y	Y
2. Quantitative (randomised controlled clinical trial, cluster or individual, randomised cross-over study)	2.1.	U										
	2.2.	U										
	2.3.	Y										
	2.4.	U										
	2.5.	Y										
3. Quantitative (non-randomised studies, e.g. non-randomised controlled trials, cohort study, case-control study, cross-sectional analytical study)	3.1.		Y	Y	Y							Y
	3.2.		Y	Y	Y							Y
	3.3.		Y	Y	Y							Y
	3.4.		Y	N	Y							Y
	3.5.		Y	Y	Y							Y
4. Quantitative (descriptive, e.g. incidence or prevalence study without a comparison group, survey, case series, case report)	4.1.				U	Y	Y	Y	Y	Y	Y	
	4.2.				Y	U	Y	U	U	U	Y	
	4.3.				Y	Y	Y	U	N	U	U	
	4.4.				U	Y	U	U	/	N	N	
	4.5.				/	Y	/	/	/	/	/	
5. Mixed methods (convergent design, sequential explanatory design, sequential exploratory design)	5.1.										U	Y
	5.2.										N	N
	5.3.										Y	Y
	5.4.										Y	N
	5.5.										N	Y

A description of each methodological quality criteria is presented in the "Mixed Methods Appraisal tool" (MMAT, 2018) Hong, Q.N., et al. Mixed methods appraisal tool (MMAT), version 2018. IC Canadian Intellectual Property Office, Industry Canada 2018; Available from http://mixedmethodsappraisaltoolpublic.pbworks.com/w/file/fetch/127916259/MMAT_2018_criteria-manual_2018-08-01_ENG.pdf.

Responses: Y - Yes; N - No; U – Unclear (Can't tell); / - Not applicable for this study.
Categories of study design: QUAN – quantitative design, QUAL – qualitative design, MIX – mixed methods design; MULTI – multimethod design.

Additional file 4. Assessment of methodological strengths and limitations.

Assessment summary - Risk of bias in individual studies

Among all studies reporting findings for CPT and IPT, more than half (57%, 50%) applied quantitative methods [8-29], followed by studies that applied a combination of methods (21%, 28%) [30-40] and those that applied qualitative methods (21%, 18%) [1-7], for CPT and IPT, respectively. Across all studies that combined quantitative and qualitative methods, most (80%) presented no methodological attempt to integrate phases, results, and data from both streams of evidence [30-32, 34-38]. Therefore, nine studies were categorised as multimethod studies [30-38] and two as mixed methods studies [39, 40]. According to the MMAT checklist, many included studies (n = 29) have methodological limitations in at least one of the quality criteria evaluated. Qualitative studies (including the qualitative component of mixed methods and multimethod studies) frequently failed to address and justify the qualitative approach applied (e.g. ethnography, phenomenology) [2, 4, 6, 30, 32, 34-38, 40]. Quantitative non-randomized studies frequently failed to address whether the intervention or exposure occurred during the study period as intended [10, 11, 15-17, 20]. Some studies also missed to provide reasons why certain eligible individuals chose not to participate [13, 17, 19]. Similarly, the risk of non-response bias was frequently not addressed across quantitative descriptive studies [24-26, 28, 29]. Overall, many studies' research question(s) lacked clarity and focus [8, 16, 29, 37-39].

We identified seven studies with methodological limitations in more than two of the quality criteria evaluated [29, 30, 36-40]. However, to gain a broader understanding of the barriers and facilitators to PT's, we did not exclude any study from our analysis regardless of the methodological limitations.

References (studies assessed using the MMAT tool)

1. Jacobson, K.B., et al., *"It's about my life": facilitators of and barriers to isoniazid preventive therapy completion among people living with HIV in rural South Africa.* AIDS Care, 2017. **29**(7): p. 936-942.
2. Lester, R., et al., *Barriers to implementation of isoniazid preventive therapy in HIV clinics: A qualitative study.* AIDS, 2010. **24**(SUPPL. 5): p. S45-S48.
3. Mugomeri, E., D. Olivier, and W.M. Van Den Heever, *Health system challenges affecting the implementation of isoniazid preventive therapy in people living with HIV in Lesotho.* HIV and AIDS Review, 2018. **17**(4): p. 299-307.
4. Okwera, A., et al., *Level of understanding of co-trimoxazole use among HIV infected, recurrent pulmonary tuberculosis suspects at a national referral tuberculosis clinic in Kampala, Uganda: a qualitative analysis.* Afr Health Sci, 2015. **15**(1): p. 49-57.
5. Selehelo, K., L. Makhado, and K.A. Madiba, *Provision of Isoniazid Preventive Therapy: The Experiences of People Living with HIV.* African Journal of Nursing and Midwifery, 2019. **21**(2).

6. Sibanda, E.L., et al., *Manuscript title: Facilitators and barriers to cotrimoxazole prophylaxis among HIV exposed babies: a qualitative study from Harare, Zimbabwe.* BMC public health, 2015. **15**: p. 784.
7. Wambiya, E.O.A., et al., *Factors affecting the acceptability of isoniazid preventive therapy among healthcare providers in selected HIV clinics in Nairobi County, Kenya: a qualitative study.* BMJ Open, 2018. **8**(12): p. e024286.
8. Chang, L.W., et al., *Effectiveness of Peer Support on Care Engagement and Preventive Care Intervention Utilization Among Pre-antiretroviral Therapy, HIV-Infected Adults in Rakai, Uganda: A Randomized Trial.* AIDS and Behavior, 2015. **19**(10): p. 1742-1751.
9. Naikoba, S., et al., *Improved HIV and TB Knowledge and Competence among Mid-level Providers in a Cluster-Randomized Trial of One-on-One Mentorship for Task Shifting.* Journal of Acquired Immune Deficiency Syndromes, 2017. **75**(5): p. e120-e127.
10. Adepoju, A.V., et al., *Implementation of Isoniazid Preventive Therapy among People Living with HIV in Northwestern Nigeria: Completion Rate and Predictive Factors.* J Glob Infect Dis, 2020. **12**(2): p. 105-111.
11. Ansa, G.A., et al., *Delivering TB/HIV services in Ghana: a comparative study of service delivery models.* Transactions of the Royal Society of Tropical Medicine and Hygiene, 2014. **108**(9): p. 560-567.
12. Khan, F.A., et al., *Performance of symptom-based tuberculosis screening among people living with HIV: not as great as hoped.* AIDS, 2014. **28**(10): p. 1463-1472.
13. Little, K.M., et al., *Predictors of isoniazid preventive therapy completion among adults newly diagnosed with HIV in rural Malawi.* Int J Tuberc Lung Dis., 2018. **22**(4): p. 371-377.
14. Louwagie, G., et al., *Missed opportunities for accessing HIV care among Tshwane tuberculosis patients under different models of care.* Int J Tuberc Lung Dis., 2012. **16**(8): p. 1052-1058.
15. Mindachew, M., et al., *Predictors of adherence to isoniazid preventive therapy among HIV positive adults in Addis Ababa, Ethiopia.* BMC public health, 2011. **11**(1): p. 916.
16. Mugomeri, E., D. Olivier, and W.M.J. van den Heever, *Tracking the rate of initiation and retention on isoniazid preventive therapy in a high human immunodeficiency virus and tuberculosis burden setting of Lesotho.* Southern African Journal of Infectious Diseases, 2019. **34**(1).
17. Munseri, P., et al., *Completion of isoniazid preventive therapy among HIV-infected patients in Tanzania.* Int J Tuberc Lung Dis., 2008. **12**(9): p. 1037-1041.
18. Ngamvithayapong, J., et al., *Adherence to tuberculosis preventive therapy among HIV-infected persons in Chiang Rai, Thailand.* AIDS, 1997. **11**(1): p. 107-112.
19. Szakacs, T.A., et al., *Adherence with isoniazid for prevention of tuberculosis among HIV-infected adults in South Africa.* BMC infectious diseases, 2006. **6**(1): p. 97.
20. Tram, K.H., et al., *Predictors of isoniazid preventive therapy completion among HIV-infected patients receiving differentiated and non-differentiated HIV care in rural Uganda.* AIDS Care, 2019. **32**(1): p. 119-127.
21. Van Ginderdeuren, E., et al., *Health system barriers to implementation of TB preventive strategies in South African primary care facilities.* PLoS ONE, 2019. **14**(2): p. e0212035.
22. Aisu, T., et al., *Preventive chemotherapy for HIV-associated tuberculosis in Uganda: an operational assessment at a voluntary counselling and testing centre.* AIDS, 1995. **9**(3): p. 267-273.
23. Chan, A.K., et al., *The Lablite project: A cross-sectional mapping survey of decentralized HIV service provision in Malawi, Uganda and Zimbabwe.* BMC health services research, 2014. **14**.
24. Faust, L., et al., *How are high burden countries implementing policies and tools for latent tuberculosis infection? A survey of current practices and barriers.* Health Sci Rep, 2020. **3**(2): p. e158.

25. Horwood, C., et al., *Prevention of mother to child transmission of HIV (PMTCT) programme in KwaZulu-Natal, South Africa: an evaluation of PMTCT implementation and integration into routine maternal, child and women's health services.* Tropical Medicine and International Health, 2010. **15**(9): p. 992-9.
26. Kamuhabwa, A.A.R. and V. Manyanga, *Challenges facing effective implementation of cotrimoxazole prophylaxis in children born to HIV-infected mothers in the public health facilities.* Drug, Healthcare and Patient Safety, 2015. **7**: p. 147-156.
27. Lai, J., et al., *Provider barriers to the uptake of isoniazid preventive therapy among people living with HIV in Ethiopia.* Int J Tuberc Lung Dis., 2019. **23**(3): p. 371-377.
28. Meribe, S.C., et al., *Sustaining tuberculosis preventive therapy scale-up through direct supportive supervision.* Public Health Action, 2020. **10**(2): p. 60-63.
29. Mwambete, K.D. and A.A. Kamuhabwa, *Resistance of commensal intestinal Escherichia coli and other enterics to co-trimoxazole and commonly used antibiotics in HIV/AIDS patients.* Clinical Microbiology: Open Access, 2013.
30. Durovni, B., et al., *The implementation of isoniazid preventive therapy in HIV clinics: the experience from the TB/HIV in Rio (THRio) study.* AIDS, 2010. **24**(Suppl 5): p. S49.
31. Gust, D.A., et al., *Risk factors for non-adherence and loss to follow-up in a three-year clinical trial in Botswana.* PLoS ONE, 2011. **6**(4): p. e18435.
32. Rowe, K.A., et al., *Adherence to TB preventive therapy for HIV-positive patients in rural South Africa: implications for antiretroviral delivery in resource-poor settings?* Int J Tuberc Lung Dis., 2005. **9**(3): p. 263-9.
33. Huerga, H., et al., *Implementation and Operational Research: Feasibility of Using Tuberculin Skin Test Screening for Initiation of 36-Month Isoniazid Preventive Therapy in HIV-Infected Patients in Resource-Constrained Settings.* JAIDS, 2016. **71**(4): p. e89-95.
34. Kamuhabwa, A.A., R. Gordian, and R.F. Mutagonda, *Implementation of co-trimoxazole preventive therapy policy for malaria in HIV-infected pregnant women in the public health facilities in Tanzania.* Drug, Healthcare and Patient Safety, 2016. **8**: p. 91-100.
35. Jarrett, B.A., et al., *Promoting Tuberculosis Preventive Therapy for People Living with HIV in South Africa: Interventions Hindered by Complicated Clinical Guidelines and Imbalanced Patient-Provider Dynamics.* AIDS and Behavior, 2019. **24**(4): p. 1106-1117.
36. Luyirika, E., et al., *Scaling Up Paediatric HIV Care with an Integrated, Family-Centred Approach: An Observational Case Study from Uganda.* PLoS ONE, 2013. **8**(8).
37. McRobie, E., et al., *HIV policy implementation in two health and demographic surveillance sites in Uganda: Findings from a national policy review, health facility surveys and key informant interviews.* Implementation Science, 2017. **12**(1).
38. Okot-Chono, R., et al., *Health system barriers affecting the implementation of collaborative TB-HIV services in Uganda.* Int J Tuberc Lung Dis., 2009. **13**(8): p. 955-961.
39. Catalani, C., et al., *A Clinical Decision Support System for Integrating Tuberculosis and HIV Care in Kenya: A Human-Centered Design Approach.* PLoS ONE, 2014. **9**(8).
40. Reddy, M.M., et al., *To start or to complete? - Challenges in implementing tuberculosis preventive therapy among people living with HIV: a mixed-methods study from Karnataka, India.* Glob Health Action, 2020. **13**(1): p. 1704540.
