# Supplementary material for: Mixed methods systematic review and metasummary about barriers and facilitators for the implementation of cotrimoxazole and isoniazid—Preventive therapies for people living with HIV
Source: PLoS One. 2022 Mar 1;17(3):e0251612. doi: 10.1371/journal.pone.0251612 (PMC8887777; doi:10.1371/journal.pone.0251612)
Supplement: S5 File — (PDF) [file pone.0251612.s005.pdf]

S5 Additional file. List of facilitators for the implementation of CPT and IPT

F1 Access: Strategies for health system strengthening, improving access to and quality of health care

Leadership & governance

- Increase the number of health care facilities (especially in rural areas) [1, 2].
- Decentralised provision of preventive therapies (PT's) and related services:
  - at health centres rather than just hospitals [3, 4], at the community level (e.g. mobile radiology units) [5].
  - home-based care for selected patient groups (i.e. HIV+ pregnant women) [6, 7].
- Raising community knowledge & awareness about HIV & PT's (via education campaigns, health talks, initiatives [8-10]:
  - to reduce stigma, peoples' fears and misperceptions (e.g. reduce the 'emasculating' stigma of HIV through education messages that emphasise how treatment after testing HIV+ can help a man achieve his 'manly' roles by maintaining his health, allowing him to 'provide for the family') [8, 9].
  - to sensitise communities about HIV testing and CPT's benefits (e.g. malaria prevention during pregnancy, prevention of opportunistic infections among babies born to HIV+ mothers) [8].
  - to increase knowledge regarding the importance of IPT, its benefits and the purpose of taking it [9, 10].
  - consider incorporating IPT into the training curriculum of undergraduate students [9].
- Encourage companies to support employee health to reduce stigma at the workplace [8].
- Strong commitment and strategic implementation support from policymakers and health authorities [10, 11]:
  - National advocacy for IPT [10].
  - Stakeholder engagement: involving policymakers, higher level supervisors and health providers in the development of policies & guidelines, in the planning of resources for monitoring, the supervision and evaluation of outcomes particularly needed for the effective implementation of IPT [10].
  - Planning for policy implementation: according to the target dimension (e.g. piloting, large scale roll-out) and ensuring additional resources depending on the complexity of the intervention (e.g. CPT<IPT) [12].
- Monitoring & Evaluation (M&E) of the implementation [12]:
  - Clearly defined, standardised indicators to evaluate the implementation progress (i.e. IPT programme) [11].
  - Decentralised responsibility of M&E (e.g. long term external oversight from district level management) [13].
  - Need for (informatic) tools and skills for routine tracking of data (e.g. initiation, coverage) [14].
  - Sharing summary data with higher-level health authorities (district team, provincial team, MoH) [14].
  - Need for a well-defined feedback mechanism of reporting indicators [10].
  - Reflecting on results, adapting the implementation process if necessary [12].
- Ensuring national and regional policies exist on:
  - Stigma reduction strategies at the patient and community level (e.g. education about HIV, TB and the concept of prevention through teaching in schools, mass media, lectures on the radio, TV, skill building for potential perpetrators of stigma, involving peers) [8].
  - Strict policies for providers on patient confidentiality and supportive service provision [15].
  - HIV service integration (with TB care, family-, well-child- & maternal health care) [8, 16-21].
  - Assessment of patients migration, travelling and relocation intentions and directions on how providers should proceed (e.g. clinic transfer, therapy prescription of >2months in advance) [18, 22].
  - Defining HIV service delivery strategy for vulnerable groups (i.e. commercial sex workers) [23].
  - Financial relief for PLHIV (subsidised transport to the clinic, free consultation, free treatment) [4, 15].
  - Social security benefits (e.g. disability grants for HIV/ AIDS, child support for caregivers of HIV+ children, nutritional support, food-partnerships with farmers, gardening or sustainable nutrition programmes) [15].
  - IPT policy modification and adequate dissemination [24].

Consider Policy update for IPT

- Considering (local, regional, national) lessons learnt for policy development of IPT delivery strategies [15, 17]
  - to identify specific target groups for scaling-up IPT (e.g. children and adolescents with HIV) [14, 25]
- Involving health providers in the development of the IPT guidelines to promote their agreement with IPT [10]
- Define set of minimum criteria to be met for IPT enrolment, such as:
  - Acceptance of HIV diagnosis [18] or openness about HIV status [15].
  - Adherent to other intensive treatment regimen (e.g. ART) [15, 18].
  - Regular clinic attendance (e.g. for at least 3 months or 4 visits) [15].
  - Counselling for patients newly diagnosed, or not yet on ART, before IPT initiation [18].
  - Requirement to attend support groups or linking HIV+ patients to community tracing [15].
- Allowing more time for patients to accept their HIV diagnosis before enrolment in intensive treatment regimen [15, 22].
- Simplifying and clarifying IPT policy [11, 13, 24, 26]:
  - Eligibility criteria; How to rule out active TB [24], role of chest radiography [24].
  - Consider using the WHO symptom screening algorithm (TST, chest radiology not mandatory) [11, 27].
  - Necessity to provide IPT together with pyridoxine [10]; monthly versus 3-monthly INH drug refills [10].
  - Health provider considerations before initiating IPT (e.g. clinical state, concurrent drug regimen) [10].

S5 Additional file. List of facilitators for the implementation of CPT and IPT

F1 Access: Strategies for health system strengthening, improving access to and quality of health care (continued).

Strengthen clinical information systems

- Allocate resources to data collection, data recording and data management (e.g. data clerks, stationery items) [5, 26].
- Update and ensure the distribution of tools (paper-based or electronic) to record IPT and patient information [5].
  - Paper-based IPT monitoring tool (that allows recording patients' TB screening information prior to and upon IPT completion, side effects, documenting last drug pickup, refill periods [28].
  - Mobile application developed by WHO or adapting the app to local needs to improve recording of IPT [4].
  - Ensure tools allow efficient recording of patient information (e.g. integrated ART/ IPT registers and prescription forms) [26, 28], patient tracing between clinics and patient loss to follow-up [12].
  - Ideally one file held per patient in semi-integrated/ integrated facilities [19, 21].
- Improve reporting accuracy [11]:
  - Clearly written instructions to guide providers in recording of clinical data [29].
  - Conducting periodic surveys of patient cards to ensure the quality of routine recording [21].
- Consider the implementation of an electronic medical record (EMR) system if the following minimum system requirements are met: adequate data quality, reliable electricity, hardware & supplies, functional printers [5].
- Monitoring of risks (i.e. adverse reactions, drug interactions) and benefits of PT's [15, 17, 18].
- Monitoring of antimicrobial resistance [30, 31].

Pharmaceutical management

- Consider the authorisation of shorter TB preventive therapy regimen recommended by WHO [10, 11].
- Consider the use of ART/INH combination therapy in one tablet [9] or combined pre-packaging of ART and INH [32].
- Strengthen central procurement & supply chain management; Empower district TB office to procure INH locally [4].
- Consider authorising dispense of INH at referral centres closer to patients' homes [4].
- Considering potential peaks of INH demand (1. during scale-up, 2. when patients turn eligible for a repeat course) [28].

Service delivery considerations

- Patient-centred approach [33]:
  - Facilitate good and trusting health provider-patient relationship [15, 18, 22].
  - Ensuring patients' confidentiality (e.g. avoiding different queues or consultation rooms for HIV services at the health centre [9], or deliver HIV services separate from the general outpatient facility [8, 15].
- Empowering patients through knowledge and education [2, 8, 9, 14, 15]:
  - General information: living healthy with HIV, the concept of prevention and that PT is taken when asymptomatic [18]. TB risk and TB prevention [6, 17, 34].
  - Information about CPT [34]: Benefits - prescribed for preventing infections or 'to strengthen patients with low immunity', discouraging self-medication and pill sharing [31], importance of taking the correct drug amount, adherence to CPT during the prescribed duration [31], CPT can be used concurrently with ART and TB treatment [34], safety of CPT and potential side effects, side effects that require interruption of CPT, and that there is an alternative medicine (dapsone) [34].
  - Information about IPT [16-18, 23, 35]: Benefits, e.g. prescribed for preventing TB and not to alleviate symptoms [11, 18], importance of IPT [1], adherence to IPT, correct administration and duration of IPT [18], concurrent use with other treatments (e.g. ART), potential side effects (some treatable with pyridoxine), side effects requiring therapy interruption [18], encouraging reporting of side effects or complaints [18]
- Consider alternative approaches to deliver PT's:
  - TB/HIV service integration (requires close relationship between TB and HIV programmes, political will and considerable routine planning, coordination and monetary efforts [10, 16, 17, 21, 25].
  - 'Directly observed therapy' (DOT) approach for IPT [9].
  - Pharmacists delivering IPT (i.e. TB symptom screening, patient education, prescription and follow-up) [32].
- Develop patient education materials and ensure their distribution and availability at the health facilities [34].
- Consider establishing an initiative that engages patients' cultural, religious and spiritual beliefs (e.g. engage traditional healers to work alongside health providers in the clinic environment) [8].

Health system financing

- Financial commitment of Ministry of Health & donors [12] to fund HIV response [12], TB/HIV service collaboration [24].
- Additional funds needed for employment of health providers [12, 26], commodities and lab facilities [12].

Strengthen health provider capacity and knowledge

- Additional employment & shifting tasks to lower cadre to scale up HIV care and PT [7, 16, 21, 29, 32, 36, 37].
  - Ensuring a reasonable patient-provider ratio [13], hiring personnel dedicated to IPT related activities [10].
  - Involving nurses [36], lay counsellors [29], pharmacists [32], social- and community workers [16], as well as PLHIV [7, 21] (i.e. peers, expert patients) to provide psychological support, PT, basic tasks, dispense tablets.
- Ensuring provider training & refresher training [3, 6, 13, 16, 17, 21, 24, 26, 29, 33, 37].
- Improving the working conditions of health providers (e.g. work environment, reduce provider workload) [10, 11].

S5 Additional file. List of facilitators for the implementation of CPT and IPT

F2	Entry to health facility: Strategies to enhance patients' willingness and feasibility to enter public healthcare

- Welcoming and safe clinic environment, friendly and supportive health providers [15, 33].
- Making visits brief & convenient (i.e. offering all services on all clinic days, adding weekend clinic hours) [2, 17].
- Ensuring patients' confidentiality to reduce the fear of stigma/ discrimination [8, 15, 29].
- Encouragement & advice from peers (post -test club, support groups) [15, 16].
- No clinic consultation fees; no dispensing fees for patients with chronic diseases (i.e. HIV) [15, 21].
- Building incentives into service delivery (e.g. subsidized transport/ reimbursement, nutritional support) [1, 15, 22, 35].
- Family & community support and encouragement (emotional, financial, organisational support) [8, 15, 18].
- Consider integrating alternative medicine, church services, and family counselling into public health care if western medicine is not widely accepted (i.e. opposed by family, friends, church leaders, traditional medicine) [15].
- Providing patient information during pre-and post-test counselling [1, 8, 16, 18] or every clinic visit [6].

F3	Eligibility: Strategies to increase health providers willingness and capability to determine patients' eligibility for PT's

- Empowering providers through training [3, 4, 6, 10, 11, 13, 15-17, 21, 24, 26, 28, 29, 33]:
  General information:
    - current epidemiology of HIV & TB [3, 17] and TB/HIV collaborative service provision [21].
    - clinical particularities of TB/HIV co-infection [17], managing complex cases [38].
    - current policies and guidelines regarding the treatment of latent TB, TB and HIV [13].
    - public health importance of detecting and treating active TB among patients with HIV [17].
    - How to facilitate nutritional support and social security grants [15].
    - Communication skills (facilitating a good and trusting provider-patient relationship) [33].

  Information on CPT:
    - CPT for HIV+ pregnant women [6, 37] and HIV-exposed babies [37].

  Information on IPT:
    - Benefits of IPT [11], importance of IPT/ treating latent TB among patients with HIV [13, 17].
    - Present current research consensus supporting IPT [17], showing IPT does not promote INH resistance [24].
    - How to rule out TB and determine if patients are eligible for IPT.
    - Increase providers’ confidence [3], capability [11] and motivation to prescribe IPT [11].
    - Managing side effects and supporting patients to complete IPT [33].
    - Difference between tuberculin and BCG vaccine (if applicable) and monitoring the tuberculin cold chain [39]
- Measures to improve health providers' acceptability of IPT [10], their confidence and motivation to prescribe IPT:
    - Mentorship [3, 9, 13, 28], supportive supervision [3, 10, 17, 28] (e.g. from district level coordinators [13]), selecting and training an 'IPT champion' [13, 24], text messaging reminders of key criteria to determine IPT eligibility [13], ensuring a consistent supply of INH and pryridoxine [3].
    - Involving local opinion leaders to change providers perception about IPT [2].
- Ensuring capacity for TB diagnosis & TB treatment essential for provision of IPT (i.e. adequate number of trained providers, facility infrastructure, ensuring a consistent supply of drugs) [3, 5, 26].
- Ensuring guidelines are well written, understandable and available at the health facility level [3, 6, 10].
- Ensuring routine evaluation and re-evaluation of patients' eligibility for IPT [9, 32].
- Availability of IPT recording tool (e.g. IPT register/ monitoring tool) [28].
- Tools to document IPT status (e.g. sticker reminder on patient file) [28].
- Tools to guide IPT decision making (e.g. paper-based work aid, or embedded electronic tool) [5].

F4	Provision: Strategies to enhance health providers' prescribing practices of PT's (particularly IPT)

- Closing providers' knowledge gaps and enhancing their attitudes towards PT (particularly regarding IPT):
    - Educational campaigns & advocacy activities that include evidence basis and involve local opinion leaders, 'IPT champions' setting an example, prescribing IPT; positively influencing attitudes toward IPT [5, 17, 24, 33].
- Visible leadership, supervision and support of health providers:
    - Roles & responsibilities in PT provision clear [15, 29].
    - Reinforcing national guidelines, provider encouragement by IPT champion and at weekly group meetings [13].
    - One-on-one mentorship & supervision with feedback to support continuous skill acquisition and health providers' confidence to deliver tasks (e.g. ruling out TB, recording patient information, prescription of PT, prepare for- and managing side effects, treatment decisions for complex cases) [3, 11, 13, 17, 18, 24, 38, 39].
    - Quality improvement approach to identify problems and take corrective action (teaching basic concept, routine data collection, chart analysis, weekly collaborative meetings to discuss successes and challenges) [28, 33].
    - Quarterly site visits for record review (e.g. organised by district TB, HIV programme managers) [9, 28].
- Empowering patients to ask for IPT (e.g. posters and health education sessions at the health facility) [13].
- Review the process of PT delivery [39], tailoring it to the clinic routine [11], considering contextual challenges [10]:
    - Participatory approach (involving health providers) [11, 39], generation and use of data, lessons learnt [28].
    - Consider reorganising services, improving patient flow within the clinic to avoid long waits [12, 15, 17].

S5 Additional file. List of facilitators for the implementation of CPT and IPT

F5	Availability: Strategies to improve the availability of PT's at public health facilities and pharmacies

- Focus on ensuring the availability of CTZ and INH [5, 9, 13, 18, 26]:
  - Improving technical and logistic capacity at health facilities [10].
  - Personnel responsible for inventory management, stock audits & the availability of stock at each level [5, 18].
  - District level initiatives to improve INH availability (e.g. monthly monitoring of INH) [13].
  - Improving supply chain management [3, 5, 10, 26, 28, 37].
  - Implementing a stock inventory management system [6], detailed accounting of stock supplies at all levels [5].
  - Quantification and demand forecasting [6]; include back-up stock (additional buffer) [5].
  - Routine stock audits to identify & address INH stock-outs at health facilities and pharmacies [35].
  - Developing stock capacities centrally, decentralised and at the health facility level [5, 40].
- Health facility approach for insufficient stock:
  - Prioritising patient subgroups (e.g. based on CD4 count, compliant with clinic visits, adherence to ART) [4].
  - Earmarking drugs for a full course of IPT (e.g. 6-months) for each patient who initiated IPT [4, 28].
- Health facility approaches in case of stock-outs:
  - Informing patients about stock-outs to avoid confusion (e.g. patient may think he/she completed PT) [18].
  - Prescribing alternative formulation of CTZ/ INH (e.g. tablets instead of suspension for children) [37].
  - Advising patients to return when stock is available [37] or buy CTZ/ INH in private pharmacies [6, 21, 31, 37].

F6	Adherence: Strategies to enhance patients willingness and feasibility to adhere to PT's

- Individual assessment of patients' needs, concerns and feasibility to initiate IPT:
  - Consider initiating IPT at the time of HIV diagnosis [25].
  - Need for providers to discuss patients' feasibility of (e.g. monthly) clinic visits, and offer PT based on individual assessment of patient's needs, concerns and commitment to follow clinic visits [18, 22, 37].
  - Discussing patients' travel plans (e.g. consider prescribing >2months of IPT for patients who will travel [18].
  - Facilitate clinic transfer between different cities/ provinces or allow treatment supply in advance [18].
- Allowing more time before enrolling patients into intensive treatment regimen (if policy & guideline allows) [15, 16] .
- Educating patients before initiating IPT [9]:
  - Risk of developing TB, importance of IPT, side effects; dapsone, pyridoxine to manage side effects [9, 18, 34].
  - Encouraging patients to report problems or complaints [18], support patients in managing side effects [9].
  - Encourage patients to disclose HIV diagnosis to a trusted person or family member [15].
- Health facility support services:
  - Support groups, nutritionist, food parcels, multivitamins [15], transport support [4, 15].
  - Couple testing [8], Involvement of partner/ friend during initiation of IPT; ensuring family approval of PT [1, 8].
- Patients' adherence support:
  - Social support or feeling comfortable taking PT in front of others [1, 6, 8, 20, 33].
  - Reminder systems developed by patients (e.g. phone alarms [8, 22], marking a calender [22]).
  - Adherence club member collects ART and INH for the group members [9].
- Health facility-based adherence support:
  - clinic visit reminders or follow-up by health providers (e.g. through phone calls, home visits to follow up and encourage defaulters/ community tracing) [3, 6, 15, 16, 22].
  - Adherence assessment (patient self-report, pill counts during treatment refills, INH metabolite testing) [6, 35].
  - Provider encouragement and follow-up to adhere to PT [3, 9].

F7	Retention in care: Strategies that encourage patients' completion of PT

- Patients self-motivation:
  - Belief that PT is useful improves health status or is important to prevent disease [1, 8, 9, 18, 33, 34].
  - Belief in personal health threat or fear of disease (e.g. concern about family) [8, 22].
  - Belief in the effectiveness of health care, understanding the importance of PT's.
- Acceptance of HIV status, disclosure to supportive person, comfortable to take PT in front of others [9, 15, 22, 23].
- Reducing patients' need to travel to multiple clinics:
  - Same-day appointments for family members [8].
  - HIV service integration with other services (e.g. providing ART + IPT; TB treatment + CPT at the same time and location) [10, 17, 18, 21, 32].
- Reducing patients' frequency of health facility visits and medication pickups:
  - Differentiated care: e.g. quarterly clinic attendance for clinically 'stable' PLHIV [18, 33].
  - Home visits or home-based care (with health facility referral of complex cases) [6, 33].
  - Adherence club member collects ART and INH for the group of members [9].
- Positive attitudes of health providers or good provider-patient relationship [9].
- Patient follow-up or linking HIV+ patients to community tracing in case they miss clinic appointments [3, 15].

References

1. Munseri P, Talbot E, Mtei L, Fordham von Reyn C. Completion of isoniazid preventive therapy among HIV-infected patients in Tanzania. Int J Tuberc Lung Dis. 2008;12(9):1037-41.
2. Gust DA, Mosimaneotsile B, Mathebula U, Chingapane B, Gaul Z, Pals SL, et al. Risk factors for non-adherence and loss to follow-up in a three-year clinical trial in Botswana. PLoS ONE. 2011;6(4):e18435.
3. Lai J, Dememew Z, Jerene D, Abashawl A, Feleke B, Teklu AM, et al. Provider barriers to the uptake of isoniazid preventive therapy among people living with HIV in Ethiopia. Int J Tuberc Lung Dis. 2019;23(3):371-7. Epub 2019/03/16. doi: 10.5588/ijtld.18.0378. PubMed PMID: 30871669; PubMed Central PMCID: PMCPMC6822021.
4. Reddy MM, Thekkur P, Ramya N, Kamath PBT, Shastri SG, Kumar RBN, et al. To start or to complete? - Challenges in implementing tuberculosis preventive therapy among people living with HIV: a mixed-methods study from Karnataka, India. Glob Health Action. 2020;13(1):1704540. Epub 2020/01/16. doi: 10.1080/16549716.2019.1704540. PubMed PMID: 31937200; PubMed Central PMCID: PMCPMC7006687.
5. Catalani C, Green E, Owiti P, Keny A, Diero L, Yeung A, et al. A Clinical Decision Support System for Integrating Tuberculosis and HIV Care in Kenya: A Human-Centered Design Approach. PLoS ONE. 2014;9(8). doi: 10.1371/journal.pone.0103205. PubMed PMID: WOS:000341127500009.
6. Kamuhabwa AA, Gordian R, Mutagonda RF. Implementation of co-trimoxazole preventive therapy policy for malaria in HIV-infected pregnant women in the public health facilities in Tanzania. Drug, healthcare and patient safety. 2016;8:91-100. Epub 2016/12/23. doi: 10.2147/dhps.s119073. PubMed PMID: 28008284; PubMed Central PMCID: PMCPmc5167525.
7. Chang LW, Nakigozi G, Billioux VG, Gray RH, Serwadda D, Quinn TC, et al. Effectiveness of Peer Support on Care Engagement and Preventive Care Intervention Utilization Among Pre-antiretroviral Therapy, HIV-Infected Adults in Rakai, Uganda: A Randomized Trial. AIDS and Behavior. 2015;19(10):1742-51. doi: 10.1007/s10461-015-1159-y.
8. Sibanda EL, Bernays S, Weller IV, Hakim JG, Cowan FM. Manuscript title: Facilitators and barriers to cotrimoxazole prophylaxis among HIV exposed babies: a qualitative study from Harare, Zimbabwe. BMC public health. 2015;15:784. Epub 2015/08/16. doi: 10.1186/s12889-015-2136-0. PubMed PMID: 26276143; PubMed Central PMCID: PMCPmc4536866.
9. Selehelo K, Makhado L, Madiba KA. Provision of Isoniazid Preventive Therapy: The Experiences of People Living with HIV. African Journal of Nursing and Midwifery. 2019;21(2). doi: 10.25159/2520-5293/6221. PubMed PMID: WOS:000532581600019.
10. Wambiya EOA, Atela M, Eboreime E, Ibisomi L. Factors affecting the acceptability of isoniazid preventive therapy among healthcare providers in selected HIV clinics in Nairobi County, Kenya: a qualitative study. BMJ Open. 2018;8(12):e024286. Epub 2018/12/24. doi: 10.1136/bmjopen-2018-024286. PubMed PMID: 30573488; PubMed Central PMCID: PMCPMC6303693.
11. Van Ginderdeuren E, Bassett J, Hanrahan C, Mutunga L, Van Rie A. Health system barriers to implementation of TB preventive strategies in South African primary care facilities. PLoS ONE. 2019;14(2):e0212035. Epub 2019/02/15. doi:

10.1371/journal.pone.0212035. PubMed PMID: 30763378; PubMed Central PMCID: PMCPMC6375590.

12. McRobie E, Wringe A, Nakiyingi-Miiro J, Kiweewa F, Lutalo T, Nakigozi G, et al. HIV policy implementation in two health and demographic surveillance sites in Uganda: Findings from a national policy review, health facility surveys and key informant interviews. Implementation Science. 2017;12(1). doi: 10.1186/s13012-017-0574-z.

13. Jarrett BA, Woznica DM, Tilchin C, Mpungose N, Motlhaoleng K, Golub JE, et al. Promoting Tuberculosis Preventive Therapy for People Living with HIV in South Africa: Interventions Hindered by Complicated Clinical Guidelines and Imbalanced Patient-Provider Dynamics. AIDS and Behavior. 2019;24(4):1106-17. doi: 10.1007/s10461-019-02675-6. PubMed PMID: WOS:000520707300012.

14. Mugomeri E, Olivier D, van den Heever WMJ. Tracking the rate of initiation and retention on isoniazid preventive therapy in a high human immunodeficiency virus and tuberculosis burden setting of Lesotho. Southern African Journal of Infectious Diseases. 2019;34(1). doi: 10.4102/sajid.v34i1.10. PubMed PMID: WOS:000519626300001.

15. Rowe KA, Makhubele B, Hargreaves JR, Porter JD, Hausler HP, Pronyk PM. Adherence to TB preventive therapy for HIV-positive patients in rural South Africa: implications for antiretroviral delivery in resource-poor settings? Int J Tuberc Lung Dis. 2005;9(3):263-9. Epub 2005/03/25. PubMed PMID: 15786888.

16. Aisu T, Raviglione MC, Eriki P, Narain J, Barugahare L, Tembo G, et al. Preventive chemotherapy for HIV-associated tuberculosis in Uganda: an operational assessment at a voluntary counselling and testing centre. AIDS. 1995;9(3):267-73.

17. Durovni B, Cavalcante SC, Saraceni V, Vellozo V, Israel G, King BS, et al. The implementation of isoniazid preventive therapy in HIV clinics: the experience from the TB/HIV in Rio (THRio) study. AIDS. 2010;24(Suppl 5):S49.

18. Jacobson KB, Niccolai L, Mtungwa N, Moll AP, Shenoi SV. "It's about my life": facilitators of and barriers to isoniazid preventive therapy completion among people living with HIV in rural South Africa. AIDS Care. 2017;29(7):936-42. doi: 10.1080/09540121.2017.1283390. PubMed PMID: WOS:000402669100019.

19. Louwagie G, Girdler-Brown B, Odendaal R, Rossouw T, Johnson S, Van der Walt M. Missed opportunities for accessing HIV care among Tshwane tuberculosis patients under different models of care. Int J Tuberc Lung Dis. 2012;16(8):1052-8. doi: 10.5588/ijtld.11.0753. PubMed PMID: WOS:000306678800012.

20. Luyirika E, Towle MS, Achan J, Muhangi J, Senyimba C, Lule F, et al. Scaling Up Paediatric HIV Care with an Integrated, Family-Centred Approach: An Observational Case Study from Uganda. PLoS ONE. 2013;8(8). doi: 10.1371/journal.pone.0069548. PubMed PMID: WOS:000324401500013.

21. Okot-Chono R, Mugisha F, Adatu F, Madraa E, Dlodlo R, Fujiwara P. Health system barriers affecting the implementation of collaborative TB-HIV services in Uganda. Int J Tuberc Lung Dis. 2009;13(8):955-61.

22. Ngamvithayapong J, Uthaivoravit W, Yanai H, Akarasewi P, Sawanpanyalert P. Adherence to tuberculosis preventive therapy among HIV-infected persons in Chiang Rai, Thailand. AIDS. 1997;11(1):107-12.

23. Mindachew M, Deribew A, Tessema F, Biadgilign S. Predictors of adherence to isoniazid preventive therapy among HIV positive adults in Addis Ababa, Ethiopia. BMC public health. 2011;11(1):916.

24. Lester R, Hamilton R, Charalambous S, Dwadwa T, Chandler C, Churchyard GJ, et al. Barriers to implementation of isoniazid preventive therapy in HIV clinics: A qualitative study. AIDS. 2010;24(SUPPL. 5):S45-S8. doi: 10.1097/01.aids.0000391021.18284.12.

25. Little KM, Khundi M, Barnes GL, Ngwira LG, Nkhoma A, Makombe S, et al. Predictors of isoniazid preventive therapy completion among adults newly diagnosed with HIV in rural Malawi. Int J Tuberc Lung Dis. 2018;22(4):371-7. Epub 2018/03/23. doi: 10.5588/ijtld.16.0836.

26. Mugomeri E, Olivier D, Van Den Heever WM. Health system challenges affecting the implementation of isoniazid preventive therapy in people living with HIV in Lesotho. HIV and AIDS Review. 2018;17(4):299-307. doi: 10.5114/hivar.2018.80263.

27. Faust L, Ruhwald M, Schumacher S, Pai M. How are high burden countries implementing policies and tools for latent tuberculosis infection? A survey of current practices and barriers. Health Sci Rep. 2020;3(2):e158. Epub 2020/05/07. doi: 10.1002/hsr2.158. PubMed PMID: 32373716; PubMed Central PMCID: PMCPMC7196590.

28. Meribe SC, Adamu Y, Adebayo-Abikoye E, Lawal I, Amazue-Ezeuko I, Okeji N, et al. Sustaining tuberculosis preventive therapy scale-up through direct supportive supervision. Public Health Action. 2020;10(2):60-3. Epub 2020/07/09. doi: 10.5588/pha.20.0003. PubMed PMID: 32639481; PubMed Central PMCID: PMCPMC7316438.

29. Horwood C, Haskins L, Vermaak K, Phakathi S, Subbaye R, Doherty T. Prevention of mother to child transmission of HIV (PMTCT) programme in KwaZulu-Natal, South Africa: an evaluation of PMTCT implementation and integration into routine maternal, child and women's health services. Tropical Medicine and International Health. 2010;15(9):992-9. Epub 2010/06/22. doi: 10.1111/j.1365-3156.2010.02576.x. PubMed PMID: 20561313.

30. Khan FA, Verkuijl S, Parrish A, Chikwava F, Ntumy R, El-Sadr W, et al. Performance of symptom-based tuberculosis screening among people living with HIV: not as great as hoped. AIDS. 2014;28(10):1463-72. doi: 10.1097/qad.0000000000000278. PubMed PMID: WOS:000337705400009.

31. Mwambete KD, Kamuhabwa AA. Resistance of commensal intestinal Escherichia coli and other enterics to co-trimoxazole and commonly used antibiotics in HIV/AIDS patients. Clinical Microbiology: Open Access. 2013.

32. Adepoju AV, Ogbudebe CL, Adejumo OA, Okolie J, Inegbeboh JO. Implementation of Isoniazid Preventive Therapy among People Living with HIV in Northwestern Nigeria: Completion Rate and Predictive Factors. J Glob Infect Dis. 2020;12(2):105-11. Epub 2020/08/11. doi: 10.4103/jgid.jgid_138_18. PubMed PMID: 32773999; PubMed Central PMCID: PMCPMC7384686.

33. Tram KH, Mwangwa F, Chamie G, Atukunda M, Owaraganise A, Ayieko J, et al. Predictors of isoniazid preventive therapy completion among HIV-infected patients receiving differentiated and non-differentiated HIV care in rural Uganda. AIDS Care. 2019;32(1):119-27. doi: 10.1080/09540121.2019.1619661. PubMed PMID: WOS:000498969900018.

34. Okwera A, Mafigiri DK, Guwatudde D, Whalen C, Joloba M. Level of understanding of co-trimoxazole use among HIV infected, recurrent pulmonary tuberculosis suspects at a national referral tuberculosis clinic in Kampala, Uganda: a qualitative analysis. Afr Health Sci. 2015;15(1):49-57. Epub 2015/04/04. doi: 10.4314/ahs.v15i1.7. PubMed PMID: 25834530; PubMed Central PMCID: PMCPmc4370121.

35. Szakacs TA, Wilson D, Cameron DW, Clark M, Kocheleff P, Muller FJ, et al. Adherence with isoniazid for prevention of tuberculosis among HIV-infected adults in South Africa. BMC infectious diseases. 2006;6(1):97.

36. Ansa GA, Walley JD, Siddiqi K, Wei X. Delivering TB/HIV services in Ghana: a comparative study of service delivery models. Transactions of the Royal Society of Tropical Medicine and Hygiene. 2014;108(9):560-7. doi: 10.1093/trstmh/tru110. PubMed PMID: WOS:000343059500009.

37. Kamuhabwa AAR, Manyanga V. Challenges facing effective implementation of cotrimoxazole prophylaxis in children born to HIV-infected mothers in the public health facilities. Drug, healthcare and patient safety. 2015;7:147-56. doi: 10.2147/dhps.s89115. PubMed PMID: WOS:000215804900017.

38. Naikoba S, Senjovu KD, Mugabe P, McCarthy CF, Riley PL, Kadengye DT, et al. Improved HIV and TB Knowledge and Competence among Mid-level Providers in a Cluster-Randomized Trial of One-on-One Mentorship for Task Shifting. Journal of Acquired Immune Deficiency Syndromes. 2017;75(5):e120-e7. doi: 10.1097/QAI.0000000000001378.

39. Huerga H, Mueller Y, Ferlazzo G, Mpala Q, Bevilacqua P, Vasquez B, et al. Implementation and Operational Research: Feasibility of Using Tuberculin Skin Test Screening for Initiation of 36-Month Isoniazid Preventive Therapy in HIV-Infected Patients in Resource-Constrained Settings. JAIDS. 2016;71(4):e89-95. Epub 2016/02/26. doi: 10.1097/qai.0000000000000895. PubMed PMID: 26910386.

40. Chan AK, Ford D, Namata H, Muzambi M, Nkhata MJ, Abongomera G, et al. The Lablite project: A cross-sectional mapping survey of decentralized HIV service provision in Malawi, Uganda and Zimbabwe. BMC health services research. 2014;14. doi: 10.1186/1472-6963-14-352. PubMed PMID: WOS:000340917700001.
